# Deep reinforcement learning–based reversible medical image encryption framework for secure IoMT environments

**DOI:** 10.3389/frai.2026.1839687

**Published:** 2026-06-16

**Authors:** K. Mahalakshmi, Sivakumar Nagarajan

**Affiliations:** 1 Department of Mathematics, School of Advanced Sciences, Vellore Institute of Technology, Vellore, Tamil Nadu, India; 2 School of Computer Science and Engineering, Vellore Institute of Technology, Vellore, Tamil Nadu, India

**Keywords:** chaotic cryptography, deep reinforcement learning, deterministic encryption, differential attack resistance, Internet of Medical Things (IoMT), medical image encryption, reversible encryption

## Abstract

The Internet of Medical Things (IoMT) environments face significant challenges in securely transmitting and storing medical images due to limited computational resources, multiple device types, and increasing cybersecurity threats. This paper describes a reversible RGB medical image encryption framework that employs deep reinforcement learning by combining adaptive policy learning with deterministic cryptographic algorithms. A Deep Q-Network (DQN) is used to dynamically select encryption actions based on statistical features extracted from the intermediate encrypted image state. To achieve strong security and precise image recovery, the framework employs a multi-layer reversible technique that comprises SHA-512-based keystream masking, Arnold scrambling with padding preservation, and chaotic diffusion. Extensive testing shows that this technique achieves high entropy, virtually optimum Number of Pixel Change Rate (NPCR) and Unified Average Changing Intensity (UACI) metrics, minimal pixel correlation and near-zero Structural Similarity Index Measure (SSIM) between the original and encrypted images, indicating a robust protection against statistical and differential attacks. Furthermore, the framework is robust against noise, data loss, occlusion, chosen plaintext, and determinism leaking attacks. Unlike fixed chaos-based encryption systems, the proposed framework introduces reinforcement learning–based adaptive action selection within a strictly reversible cryptographic pipeline. The effective key space exceeds 2^512^ due to SHA-512–based seed derivation and nonce-driven randomness. The overall computational complexity of the encryption process is O(H × W × T), making it scalable for high-resolution medical images. Experimental results demonstrate entropy values approaching the theoretical maximum (7.999), NPCR above 99.9%, and UACI up to 40%, confirming strong diffusion and resistance against differential and chosen-plaintext attacks.

## 1 Introduction

In contemporary healthcare, one of the primary uses of medical imaging data is diagnosis and record-keeping. Along with the growth of the internet-based systems, it has become crucial to guarantee the security of medical data that is electronic in nature, both during storage and during its transmission. Secure exchange of medical data, which is a significant aspect of telemedicine, particularly in teleradiology, where medical images like X-rays, CT-scans, and MRIs are sent through the networks for analysis by experts in remote locations (Belazi and Mabrouk, 2025). To maintain the anonymity of the patients, these images are usually encrypted, although the traditional encryption algorithms treat them as streams or blocks of binary bits (Zhang et al., 2022). The COVID-19 pandemic in 2019 led to the creation and sharing of large volumes of medical imaging and electronic health records online every day (Almeida et al., 2020). The privacy issue became a huge concern very quickly because of this rapid growth, since the medical data was of the utmost sensitivity and would be easily accessible if unauthorized persons got into the Internet of Medical Things (IoMT) environment. IoMT devices often operate under limited processing power and memory constraints. Besides, they have different architectures, protocols, and security levels, all making the use of strong encryption techniques difficult. Moreover, IoMT devices linked to open networks that have medical record storage systems usually do not update their firewalls, and thus they become susceptible to privilege-escalation attacks (He et al., 2024). The security weaknesses of a system can be effectively reduced through routine firewall updates (Rasool et al., 2022). Advanced medical image encryption techniques have been developed by researchers to address these security challenges. Recent research addresses medical image security. Yasser et al. (2021) worked on a chaos-based encryption technique. El-Shafai et al. (2022) put forward a secure optical cryptosystem. Alqahtani et al. (2022) explored fractional Fourier transforms for image recognition. Khatib et al. (2024) introduced an encryption method using Chen hyper-chaotic systems. Zhou et al. (2022) built a color image encryption system using LSTM networks to train chaotic signals for encryption. Recent work shows that deep learning is becoming more useful for image security and analysis, alongside older methods like chaos and cryptography. Deep learning excels at automatic feature extraction from images, leading to its use in tasks like finding copy-move forgeries (Rao and Ni, 2016), steganalysis (Ye et al., 2017), spotting Wi-Fi impersonation (Aminanto and Kim, 2016), protecting privacy (Phong et al., 2017), and creating fuzzy theories (Kumar et al., 2017). Despite their strong diffusion characteristics, most chaos-based and learning-based encryption schemes rely on fixed transformation sequences that lack adaptability to image-specific statistical properties. Moreover, several deep learning-based encryption approaches compromise strict reversibility or introduce computational overhead unsuitable for resource-constrained IoMT devices. Therefore, there remains a need for an adaptive, fully reversible, and computationally efficient encryption framework that integrates intelligent policy learning with deterministic cryptographic guarantees. Standard chaos theory-based encryption algorithms use predefined transformation sequences that do not provide optimal solutions for various image statistical patterns. However, fixed transformation schemes fail to adapt to varying image characteristics, resulting in suboptimal diffusion and confusion performance across different inputs. Medical images exhibit diverse statistical properties such as entropy, variance, and structural complexity, which cannot be effectively handled by a single predefined transformation sequence. Therefore, an adaptive mechanism is required to dynamically select appropriate encryption operations based on the current state of the image. Reinforcement learning provides such a capability by learning an optimal action selection policy that maximizes cryptographic strength metrics during the encryption process. The system uses reinforcement learning algorithms to pick transformation options based on statistical data, resulting in increased diffusion performance while retaining full reversibility. This study proposes a secure medical image encryption architecture that offers complete reversibility and dynamic medical image protection. The proposed method uses a reinforcement learning (RL) agent which selects encryption operations according to state features that it derives from a partially encrypted image. The operations include a SHA-512-based keystream masking system that preserves both Arnold scrambling and padding together with chaotic diffusion in a deterministic multi-layer encryption system. The framework achieves complete reversibility while it enhances statistical unpredictability and improves its protection against differential attacks and structural attacks.

The main findings of this study can be summarized using the following points :

- Reinforcement-learning-based encryption: A reinforcement learning system uses dynamic encryption path selection to protect image data.
- Reversible multi-layer encryption framework: The reversible multi-layer encryption framework enables reversible encryption through lightweight operations that leverage reinforcement learning, SHA-512 key stream masking, Arnold scrambling, and chaotic diffusion.
- Adaptive action selection: Uses learning techniques to select actions that improve security against known-plaintext, chosen-plaintext, and brute-force attacks, thereby replacing traditional encryption methods.
- Improved security Performance: Three assessment methods showed higher entropy, NPCR, and UACI values than standard chaos-based systems, while maintaining performance despite occlusion and noise interruptions.
- IoMT-Friendly Design: The proposed security framework enables IoMT devices to operate with constrained system resources by managing security and computational needs.

Paper Organization: The remainder of this paper is structured as follows. Section 2 presents related work. In Section 3, the proposed method is described. Section 4 presents the security analysis and experimental finding. Discussions are presented in Section 5. The paper concludes in Section 6. Limitations and future work are described in Sections 7 and 8, respectively.

## 2 Related work

This section reviews existing medical image encryption techniques and highlights their effectiveness in ensuring data security and privacy. Prior studies employ various evaluation metrics, including statistical, differential, and computational analyses, to assess encryption performance. These works demonstrate the importance of secure encryption schemes that not only protect sensitive medical data but also ensure accurate and complete data recovery.

### 2.1 Chaos-based and cryptographic methods

Chaos-based and cryptographic methods utilize chaotic maps and classical cryptographic techniques to achieve strong confusion and diffusion. Khalid et al. (2022) developed an ECIES-based encryption system using Diffie–Hellman key exchange and SHA-256 hashing to ensure confidentiality and authentication. Similarly, Zia et al. (2022) investigated chaos-based cryptosystems across spatial, temporal, and spatiotemporal domains, demonstrating strong performance in terms of security metrics and computational efficiency. Furthermore, Bhattacharjee et al. (2023) proposed a logistic map-based encryption technique with improved time complexity and security performance, while El-Damak et al. (2024) enhanced robustness by integrating hyperchaos and Galois field operations, achieving a large key space and resistance against noise and occlusion attacks. In addition, Bashir et al. (2022) introduced an ECC-based encryption scheme combined with a 4D chaotic system to improve diffusion and resistance against plaintext attacks. DNA-chaos-based approaches have also been explored for secure medical data protection in electronic health records (Aashiq Banu et al., 2025). However, these methods generally rely on fixed transformation sequences, limiting adaptability to varying image characteristics.

### 2.2 Hybrid and multi-layer encryption methods

Hybrid encryption methods combine multiple techniques to enhance robustness and security. Luo et al. (2019) integrated elliptic curve ElGamal encryption with chaos and SHA-based key generation to improve randomness and resistance against plaintext attacks. Similarly, Deb et al. (2019) and Masood et al. (2022) proposed multi-layer encryption frameworks incorporating logistic maps, scrambling mechanisms, and cryptographic primitives to strengthen diffusion and security for multimedia data. In addition, Zhang et al. (2026) introduced a hybrid scheme using hyper-chaotic systems and finite field operations with SHA-based dynamic key generation, achieving high entropy, strong NPCR/UACI performance, and lossless reconstruction. Furthermore, Manikandan and Amirtharajan (2022) focused on DICOM-based encryption by integrating patient identity for secure access control, while Inam et al. (2024) combined LSB, chaotic maps, and DNA encoding to ensure secure data embedding with high image quality. Benssalah and Rhaskali (2020) also proposed a hybrid scheme integrating elliptic curve cryptography, Hill cipher, and Arnold mapping to enhance confusion and diffusion properties. However, these hybrid approaches still rely on predefined transformation sequences and lack adaptability to varying image characteristics.

### 2.3 Deep learning-based encryption methods

Deep learning-based methods have gained attention for their ability to learn complex patterns and enhance encryption performance. Long et al. (2024) proposed a deep learning-based encryption framework where logistic map-generated keys are combined with extracted image features to improve diffusion, while a feedback neural network enhances reconstruction quality. Similarly, Abdellatef et al. (2024) developed a Convolutional Neural Network (CNN)-based approach for secure CT image encryption by integrating feature extraction with chaotic mapping to preserve patient privacy. Furthermore, Mehmood et al. (2024) explored various machine learning and deep learning-based cryptographic techniques, including adversarial networks, deep hashing, and chaotic neural networks. Selvakumar et al. (2024) introduced a GAN-based encryption method capable of learning from unpaired datasets, making it suitable for medical imaging scenarios where labeled data is limited. However, these methods often introduce higher computational overhead and may not guarantee strict reversibility.

### 2.4 Research gap and motivation

Current image encryption algorithms based on chaotic and deep learning techniques largely rely on predefined transformation sequences, which limit adaptability and may hinder complete data restoration. Although these methods achieve strong diffusion and security, several limitations remain. Chaos-based and hybrid approaches lack adaptability to varying image characteristics, while deep learning-based methods often introduce higher computational overhead and do not guarantee strict reversibility. Moreover, existing studies have not effectively integrated adaptive reinforcement learning with reversible cryptographic mechanisms to ensure deterministic and lossless security in IoT environments. These limitations highlight the need for an adaptive, efficient, and fully reversible encryption framework, which motivates the proposed deep reinforcement learning–based approach.

## 3 Proposed method

This section describes a medical image communication system that employs deep reinforcement learning (DRL) to generate a safe reversible Red Green Blue (RGB) image encryption system that retains security, complete reversibility, and system strength. The proposed model combines a deep Q-network (DQN) with a multi-stage cryptographic pipeline that incorporates SHA-512-based masking, Arnold scrambling with padding preservation, and chaotic diffusion to provide reversible and deterministic encryption techniques.

### 3.1 Methodology overview

To improve clarity and reproducibility, the overall workflow of the proposed DRL-based reversible medical image encryption framework is summarized as follows:

1. Input Image Acquisition: The medical image (RGB or grayscale) is acquired and standardized.
2. Feature Extraction: Statistical features such as entropy, mean intensity, edge density, and variance are extracted to construct the state vector.
3. RL-Based Action Selection: The Deep Q-Network (DQN) selects an adaptive encryption action based on the current image state.
4. Multi-Layer Encryption: The selected action is followed by SHA-512-based masking, Arnold scrambling, and chaotic diffusion operations.
5. Metadata Storage: Encryption parameters, including actions, seeds, padding values, and scrambling rounds, are stored to ensure reversibility.
6. Encrypted Output: The final encrypted image is generated after completing all transformation stages.
7. Reversible Decryption: The original image is reconstructed using inverse operations and the stored metadata.

### 3.2 Overall framework

The proposed encryption system utilizes RGB medical images and consists of two major components:

1. DQN-Driven Adaptive Encryption Controller:
  A trained deep Q-network (DQN) receives a statistical state vector extracted from the current image state and outputs an adaptive encryption action *a*_*t*_. The action-selection process is guided by a reward function designed to maximize cryptographic strength metrics such as entropy, NPCR, UACI, and perceptual dissimilarity.
2. Deterministic Multi-Layer Cryptographic Transformation Pipeline:
  The selected action is executed within a reversible transformation pipeline consisting of SHA-512-based keystream masking, Arnold scrambling with padding preservation, and chaos-driven diffusion. All cryptographic operations are deterministic and parameterized using seeds derived from the original image and the encryption step index, thereby ensuring complete reversibility during decryption.

**Figure 1:**
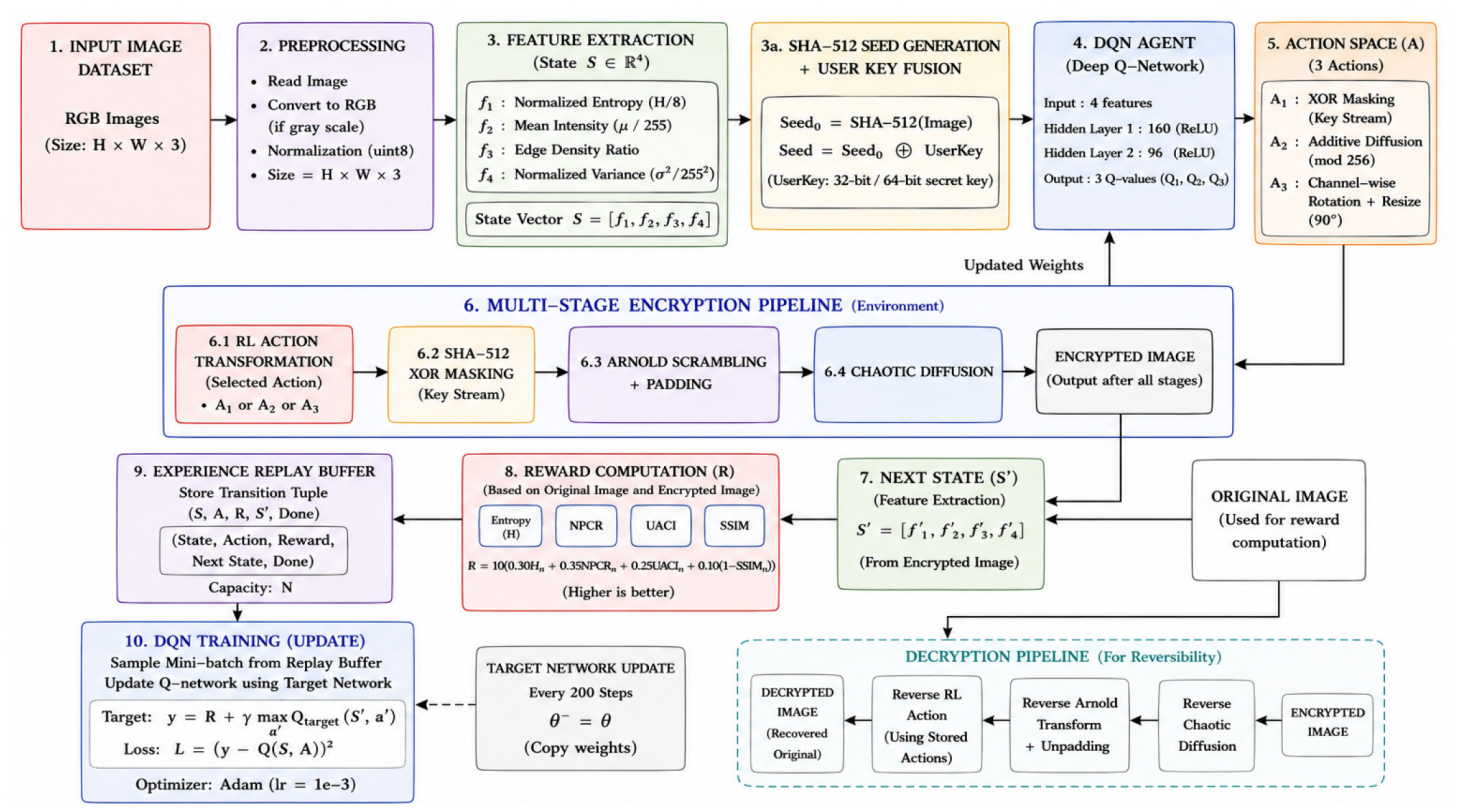
Architecture of the proposed DRL-based reversible medical image encryption framework.

Figure 1 illustrates the overall architecture of the proposed DRL-based reversible medical image encryption framework. The system consists of image preprocessing, feature extraction, DQN-based adaptive action selection, multi-stage encryption, reward computation, experience replay, and DQN training modules. The selected encryption action is applied through SHA-512 masking, Arnold scrambling, and chaotic diffusion to generate the encrypted image. The framework also supports reversible decryption using stored metadata and inverse operations.

### 3.3 Image pre-processing and feature extraction

Let *I* ∈ ℝ^*H*×*W*×3^ denote the RGB medical image with height *H* and width *W*. If the input image is grayscale, it is replicated across three channels to enable RGB processing. All images are represented using 8-bit unsigned integer intensity values in the range [0, 255].

To enable adaptive action selection, the reinforcement learning agent operates on a compact statistical representation of the current encrypted image state. At each encryption step, four statistical features are extracted from the grayscale version of the image to construct the state vector.

Let *I*_*g*_ denote the grayscale representation of the image. The four - dimensional state vector is defined as


$$\text{s}=\left[H_{n},M_{n},ER,V\right]$$

where the components are defined as follows.

#### 3.3.1 Normalized entropy

The Shannon entropy of the grayscale image is given by


$$H\left(I_{g}\right)=-\overset{255}{\underset{k=0}{\sum }}p_{k}\log_{2}\left(p_{k}\right),$$

where *p*_*k*_ denotes the probability of intensity level *k*.

The normalized entropy is defined as


$$H_{n}=\frac{H\left(I_{g}\right)}{8}$$

Because the maximum entropy of an 8-bit image is 8, this normalization ensures *H*_*n*_ ∈ [0, 1].

#### 3.3.2 Mean intensity


$$M=\frac{1}{HW}\overset{H}{\underset{x=1}{\sum }}\overset{W}{\underset{y=1}{\sum }}I_{g}\left(x,y\right)$$

To maintain scale consistency, mean intensity is normalized as


$$M_{n}=\frac{M}{255}$$

#### 3.3.3 Edge density ratio

Edge density is computed using Canny edge detection:


$$ER=\frac{\sum_{x,y}E\left(x,y\right)}{HW}$$

where *E*(*x, y*) is a binary edge map obtained using the Canny operator. This feature captures the structural complexity of the image.

#### 3.3.4 Normalized variance

The intensity variance is defined as


$$Var\left(I_{g}\right)=\frac{1}{HW}\underset{x,y}{\sum }{\left(I_{g}\left(x,y\right)-M\right)}^{2}$$

It is normalized as


$$V=\frac{Var\left(I_{g}\right)}{255^{2}}$$

The resulting feature vector **s** ∈ ℝ^4^ captures both statistical randomness (entropy and variance) and structural information (edge density), thereby enabling the DQN to adaptively select encryption actions that maximize diffusion strength and perceptual dissimilarity.

### 3.4 Deep Q-Network (DQN) design

A deep Q-network is employed to implement the reinforcement learning agent that is responsible for adaptive encryption action selection. The network architecture consists of the following components:

- Input layer: Four neurons corresponding to the extracted state features.
- Hidden layers: Two fully connected layers containing 160 and 96 neurons, respectively, each followed by a ReLU activation function.
- Output layer: Three neurons representing the available encryption actions.

The possible encryption actions are:

- XOR-based keystream masking
- Additive modular keystream diffusion
- Dimension-preserving channel-wise rotation

The agent is trained using an ϵ-greedy policy to balance exploration and exploitation. Learning stability is achieved using experience replay and a target network.

#### 3.4.1 Reward function

The reward function is designed to encourage strong cryptographic properties and perceptual dissimilarity between the original and encrypted images. It is defined as:


$$\begin{array}{l} R=10(0.30H_{n}+0.35NPCR_{n}\\ \;+0.25UACI_{n}+0.10\left(1-SSIM\right)) \end{array}$$

The weighting coefficients were selected based on the relative importance of cryptographic security metrics. In particular, higher weights are assigned to NPCR and UACI as they directly measure resistance to differential attacks and diffusion strength, which are critical in secure image encryption. Entropy is also given significant importance to ensure statistical randomness, while SSIM is assigned a lower weight as it serves as a secondary perceptual similarity measure.

A sensitivity analysis was conducted by varying the weight combinations within a reasonable range. The results indicate that emphasizing NPCR and UACI improves diffusion performance, whereas increasing entropy weight alone does not significantly enhance resistance to attacks. The selected combination (0.30, 0.35, 0.25, 0.10) provides a balanced trade-off among all evaluation metrics.

The results in Table 1 demonstrate that the selected weight configuration achieves the best balance between entropy, NPCR, and UACI, thereby validating the effectiveness of the designed reward function.

**Table 1:** Weight sensitivity analysis of reward function.

Weights (Entropy, NPCR, UACI, SSIM)	Entropy	NPCR (%)	UACI (%)
(0.25, 0.25, 0.25, 0.25)	7.89	99.71	34.12
(0.30, 0.35, 0.25, 0.10)	7.99	99.87	40.74
(0.40, 0.30, 0.20, 0.10)	7.95	99.75	36.85

The normalized metrics are defined as:


$$H_{n}=\frac{H}{8},\;NPCR_{n}=\frac{NPCR}{100},\;UACI_{n}=\frac{UACI}{33}$$

Here, *H*_*n*_ denotes the normalized entropy obtained by dividing the entropy value *H* by 8. The metrics *NPCR*_*n*_ and *UACI*_*n*_ are normalized according to their theoretical baselines, where NPCR approaches 99.6% and the grayscale UACI approaches 33.463%. The structural similarity index (SSIM) measures the similarity between the original and encrypted images at intermediate stages.

Rewards are generated after each encryption step to guide the adaptive action selection process. The weighting coefficients emphasize diffusion strength through the NPCR and UACI while maintaining statistical unpredictability and perceptual dissimilarity. A scaling factor is included to maintain numerical stability during the Q-value updates. As the objective of encryption is to maximize perceptual dissimilarity rather than reconstruction accuracy, PSNR is not included in the reward formulation. The normalization baseline of 33 corresponds to the theoretical UACI value for grayscale images; however, higher UACI values may arise in RGB-based encryption due to multi-channel diffusion effects.

The use of reinforcement learning in this framework is motivated by the need for adaptive transformation selection. Unlike fixed encryption pipelines, which apply a predefined sequence of operations, the DQN agent learns an optimal policy that dynamically selects encryption actions based on the statistical properties of the intermediate image. This enables improved diffusion and stronger resistance against statistical and differential attacks.

Table 2 summarizes the training configuration used for the proposed adaptive encryption system. With a learning rate of 0.001 and a discount factor (γ) of 0.99, the Adam optimizer was used to train the deep Q-network. A replay buffer with a capacity of 10,000 records was employed to enable stable learning through mini-batch sampling of 64 transitions. The ϵ-greedy exploration strategy gradually reduces the exploration probability from 1.0 to 0.05 with a decay rate of 0.001. The network architecture, consisting of two fully connected hidden layers with 160 and 96 neurons using ReLU activation, enables the extraction of nonlinear feature representations and facilitates adaptive encryption action selection.

**Table 2:** DQN training configuration for the proposed adaptive encryption system.

Parameter	Value
Learning rate	0.001
Discount factor (γ)	0.99
Batch size	64
Replay buffer size	10,000
Number of episodes	200
Optimizer	Adam
Hidden layer sizes	160 and 96 neurons
Action space size	3
State feature dimension	4
Initial epsilon	1.0
Minimum epsilon	0.05
Epsilon decay rate	0.001

### 3.5 Deterministic multi-layer encryption process

For each encryption step *t*, the following procedures are performed sequentially in a deterministic manner.

#### 3.5.1 RL-based action execution

The DQN selects an action *a*_*t*_ based on the current state *s*_*t*_. A deterministic keystream seed is generated from the SHA-512 hash of the original image combined with the step index. The SHA-512 output provides a 512-bit cryptographic key space, ensuring strong resistance against brute-force attacks.

The selected action is applied to the current image:


$$I_{1}=\text{ApplyAction}\left(I_{\text{cur}},a_{t},\text{seed}\right)$$

#### 3.5.2 SHA-512-based keystream masking

A SHA-512-derived seed initializes an *xorshift32* pseudo-random generator to produce a keystream. Bitwise XOR masking is then applied as follow:


$$I_{2}=I_{1}⊕K_{\text{SHA}}$$

This stage enhances the confusion property of the encryption process.

#### 3.5.3 Chaotic diffusion

A second deterministic keystream derived from the original seed and step index is used to perform feedback-based diffusion, as defined in Equation 1:


$$\begin{array}{c} I_{3}\left(i\right)=\left(I_{2}\left(i\right)+K_{\text{chaos}}\left(i\right)+I_{3}\left(i-1\right)\right)\;\mathrm{mod}\;256 \end{array}$$ (1)

where *I*_2_(*i*) is the pixel value after masking and scrambling, *K*_chaos_(*i*) is the keystream value, and *I*_3_(*i* − 1) is the previously diffused pixel. The initial condition is defined as *I*_3_(0) = *K*_chaos_(0).

The keystream *K*_chaos_ is generated from a deterministic seed obtained via the SHA-512 hash of the original image combined with the current encryption step index. Pixels are processed in a row-wise scan order, ensuring that changes propagate through subsequent pixels.

The feedback term introduces strong inter-pixel dependency, producing an avalanche effect and significantly improving resistance to differential and statistical attacks, which is reflected in high NPCR and UACI values. The diffusion process is fully reversible due to the deterministic keystream generation and modular arithmetic operations, enabling exact reconstruction during decryption.

#### 3.5.4 Arnold scrambling with padding preservation

If the image is non-square, zero-padding is applied to form an *N* × *N* matrix. The Arnold transform is then applied for a deterministic number of rounds:


$$\left(\begin{array}{c} x^{\prime }\\ y^{\prime } \end{array}\right)=\left(\begin{array}{cc} 1 & 1\\ 1 & 2 \end{array}\right)\left(\begin{array}{c} x\\ y \end{array}\right)\;\mathrm{mod}\;N$$

Padding information and the number of transformation rounds are stored to ensure perfect reversibility during decryption.

The final encrypted image is defined as


$$I_{\text{enc}}=I_{\text{cur}}$$

The complete encryption procedure of the proposed DRL-based reversible RGB image encryption framework is detailed in Algorithm 1

Algorithm 1 RL-driven reversible RGB encryption.

**Input:** Original RGB image *I*_*orig*_, trained DQN model θ, max steps *T*, Arnold limits (*R*_*min*_, *R*_*max*_)
**Output:** Encrypted image *I*_*enc*_, metadata *M*
*I*_*cur*_ ← *I*_*orig*_
*state* ← ExtractFeatures(*I*_*cur*_)
Initialize metadata *M*
**for** *t* = 1 to *T* **do**
  *seed* ← SHA512Seed(*I*_*orig*_, *t*)
  *DQN-based action selection*
  *a* ← argmax*Q*(*state*|θ)
  *Step 1: RL-controlled transform*
  *I*_1_ ← ApplyAction(*I*_*cur*_, *a, seed*)
  *Step 2: SHA-512 keystream masking*
  *I*_2_ ← SHA512KeystreamMask(*I*_1_, *seed*)
  *Step 3: Arnold scrambling with padding*
  *rounds* ← ComputeArnoldRounds(*seed, R*_*min*_, *R*_*max*_)
  (*I*_3_, *padInfo*) ← ArnoldScramble(*I*_2_, *rounds*)
  **Step 4: Chaotic diffusion (feedback-based modular diffusion)**
  *I*_cur_ ← ChaoticDiffuse(*I*_3_, seed)      ⊳
*I*_3_(*i*) = (*I*_2_(*i*) + *K*_chaos_(*i*) + *I*_3_(*i* − 1)) mod 256
  Store metadata
  *M*.*actions*[*t*] ← *a*
  *M*.*seeds*[*t*] ← *seed*
  *M*.*rounds*[*t*] ← *rounds*
  *M*.*padInfo*[*t*] ← *padInfo*
  *state* ← ExtractFeatures(*I*_*cur*_)
**end for**
*I*_*enc*_ ← *I*_*cur*_
**return** (*I*_*enc*_, *M*)

### 3.6 Metadata management and reversibility

To guarantee strict reversibility, the metadata structure *M* stores the following information:

- The sequence of selected actions *a*_*t*_
- Deterministic seeds for each encryption step
- Arnold scrambling rounds
- Padding information
- Original image dimensions

This metadata ensures the exact inversion of every transformation stage during the decryption process.

### 3.7 Deterministic reversible decryption

The decryption procedure applies the inverse transformations in the reverse order of the encryption process:

- Reverse Arnold scrambling and remove padding
- Reverse chaotic diffusion
- Reverse SHA-512 masking
- Reverse RL-selected action

**Figure 2:**
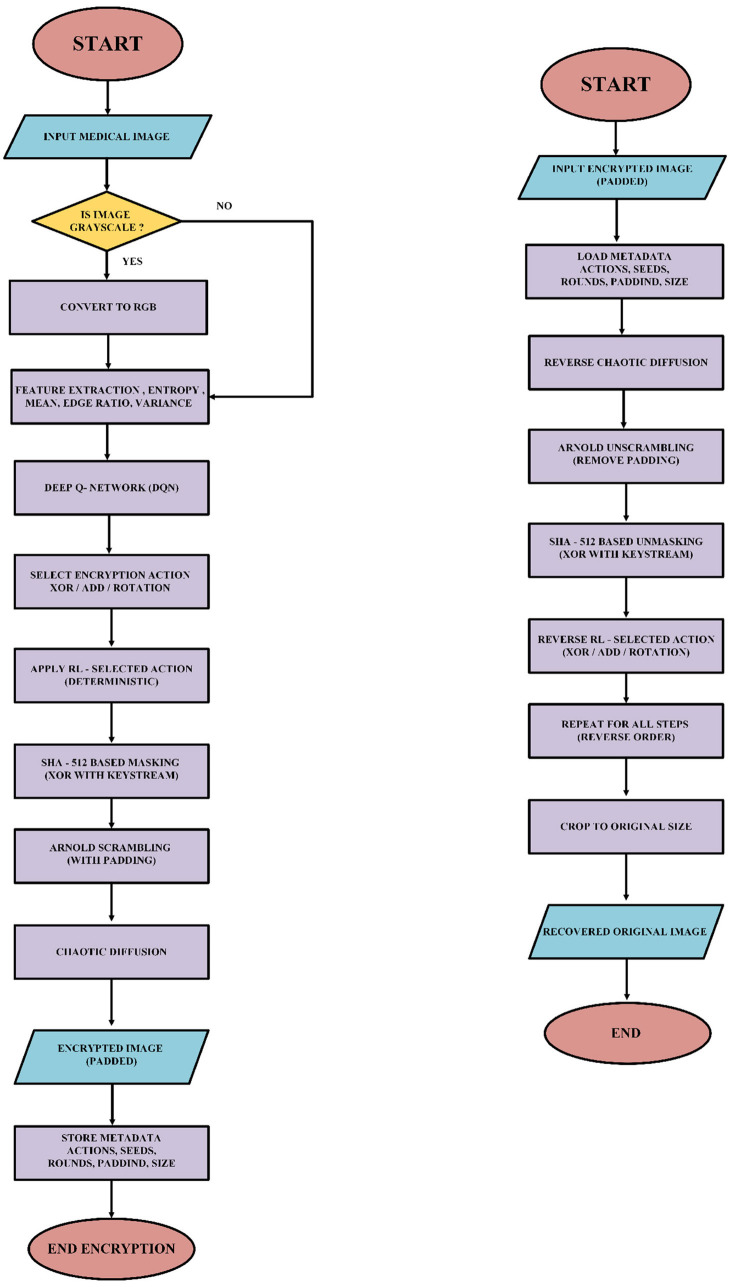
Flowchart of the proposed algorithm.

After completing all decryption steps, the image is cropped to its original dimensions, thus realizing perfect pixel-level reconstruction. The complete decryption procedure is detailed in Algorithm 2. Figure 2 shows the flowchart of the proposed encryption framework.

Algorithm 2 Deterministic reversible decryption.

**Input:** Encrypted padded image *I*_*encPad*_, metadata *M*
**Output:** Decrypted image *I*_*dec*_
*I*_*cur*_ ← *I*_*encPad*_
*T* ← length(*M*.*actions*)
**for** *t* = *T* down to 1 **do**
  *seed* ← *M*.*seeds*[*t*]
  *rounds* ← *M*.*rounds*[*t*]
  *padInfo* ← *M*.*padInfo*[*t*]
  *a* ← *M*.*actions*[*t*]
  *Step 1: Reverse chaotic diffusion*
  *I*_1_ ← ReverseChaoticDiffuse(*I*_*cur*_, *seed*)
  *Step 2: Reverse Arnold scrambling with padding removal*
  *I*_2_ ← ArnoldUnscramble(*I*_1_, *rounds, padInfo*)
  *Step 3: Reverse SHA-512-based keystream masking*
  *I*_3_ ← ReverseSHA512KeystreamMask(*I*_2_, *seed*)
  **Step 4: Chaotic diffusion (feedback-based modular diffusion)**
  *I*_cur_ ← ChaoticDiffuse(*I*_3_, seed)      ⊳
*I*_3_(*i*) = (*I*_2_(*i*) + *K*_chaos_(*i*) + *I*_3_(*i* − 1)) mod 256
**end for**
*I*_*dec*_ ← TrimToOriginalDimensions(*I*_*cur*_)
**return** *I*_*dec*_

## 4 Experimental results and security analysis

### 4.1 Experimental setup

**Figure 3:**
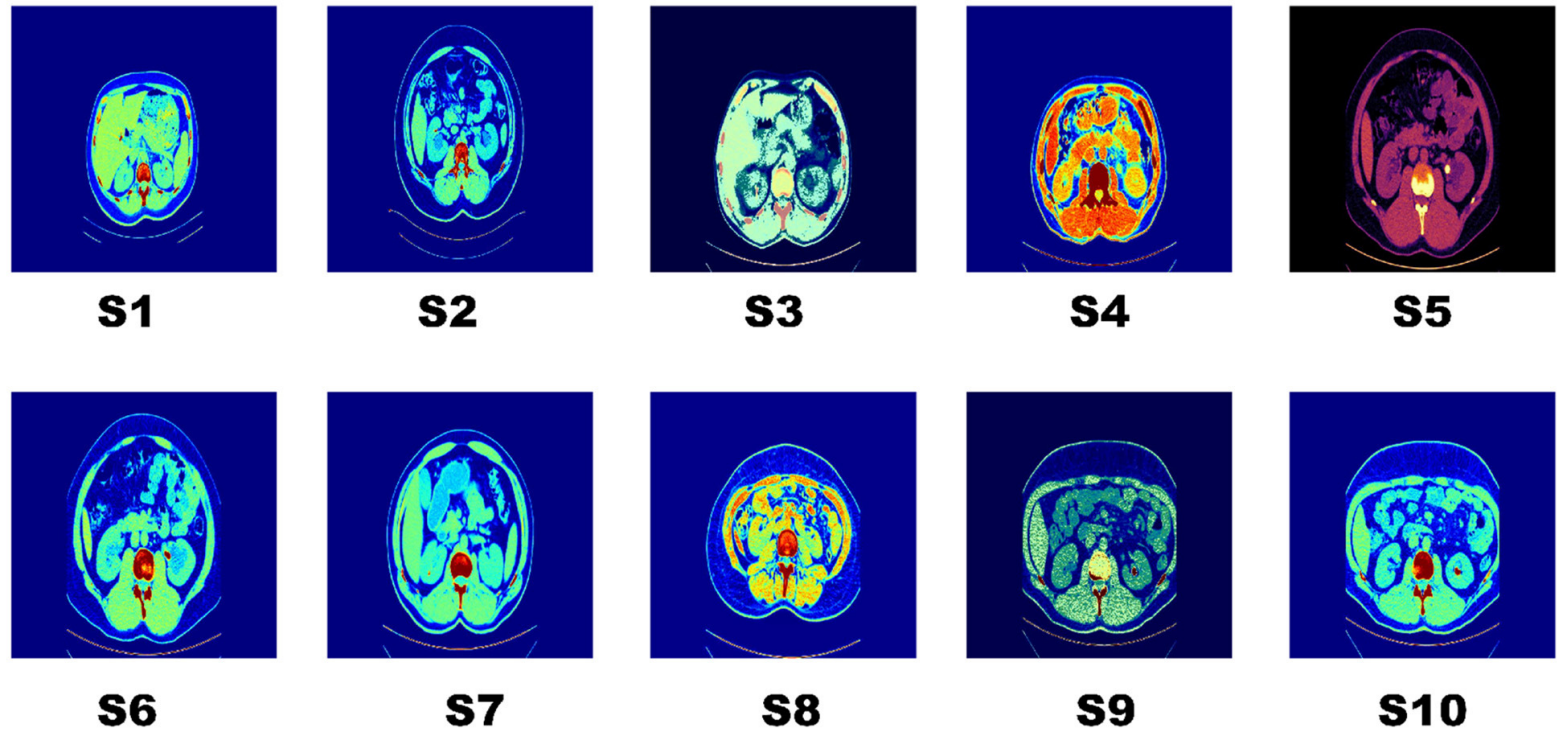
Sample medical images: S1–S10 represent the ten test images used in the experimental evaluation.

All encryption and decryption tests in MATLAB R2025a used a 1.30 GHz Intel(R) Core 12th Gen i5-1235U CPU with 16 GB of RAM. We selected multiple medical images from the Kidney Stone Axial CT Imaging Colorized Mixed Data set to evaluate the proposed encryption framework (Basak, 2023). The image files are labeled S1, S2, S3, S4, S5, S6, S7, S8, S9, and S10 and Figure 3 depicts the original sample images.

### 4.2 DQN training and policy convergence analysis

This subsection examines the DQN-based encryption controller learning process through three main aspects including training stability and policy convergence, and cryptographic strength maintenance.

#### 4.2.1 Entropy stability during training

**Figure 4:**
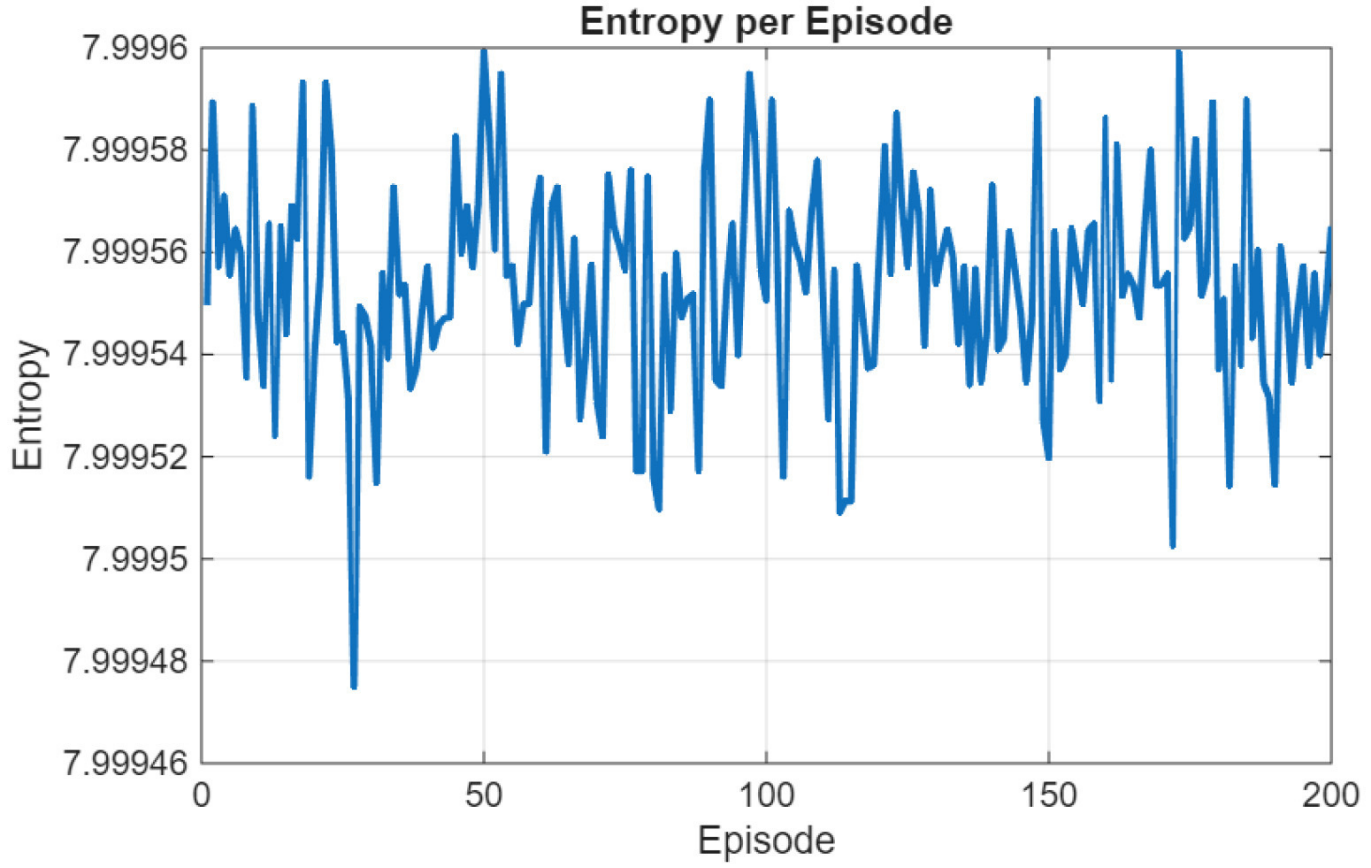
Entropy episode.

Figure 4 shows that the entropy of the encrypted images remains consistently close to the theoretical maximum, indicating that strong randomness is stable during training.

#### 4.2.2 TD loss curve

**Figure 5:**
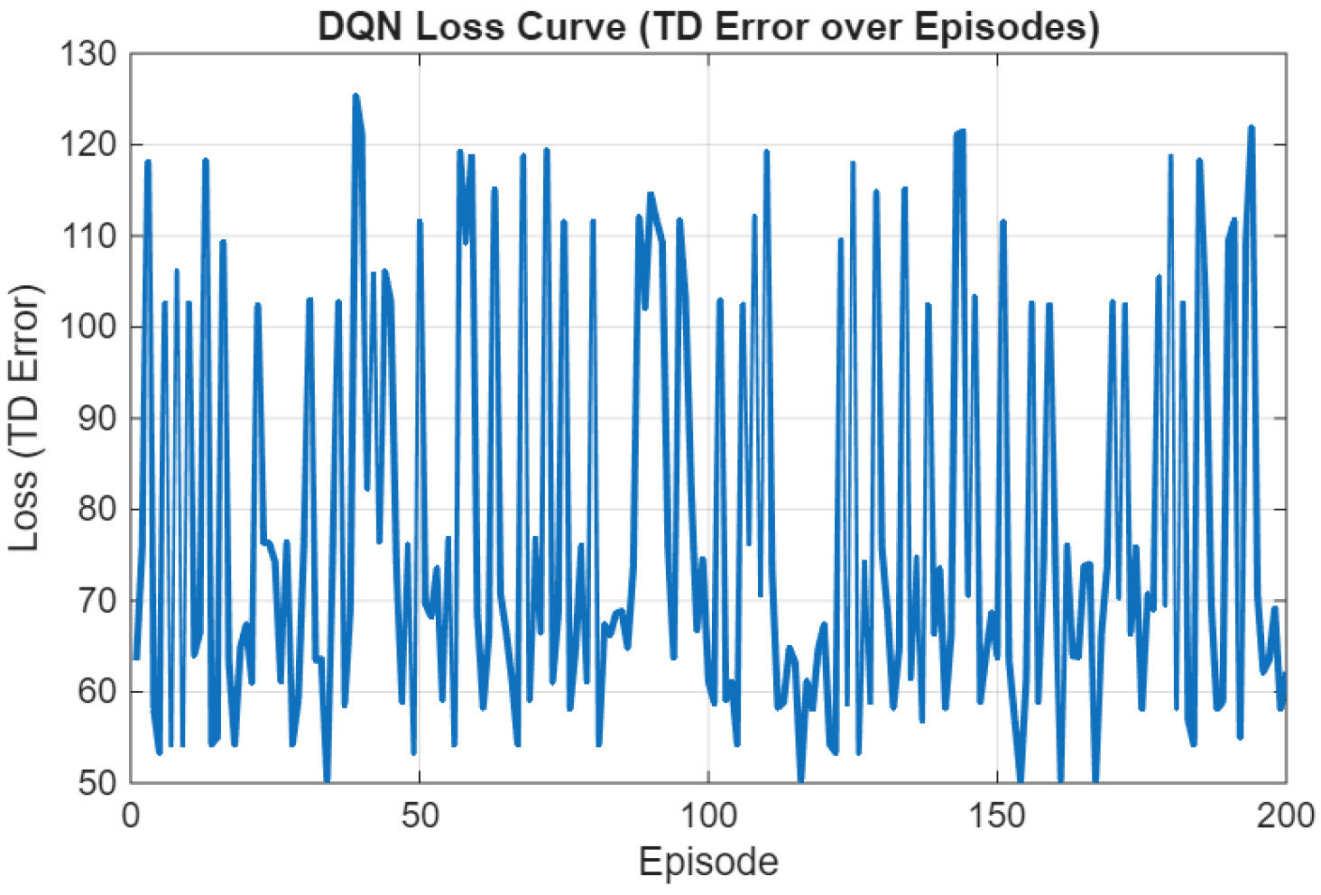
Loss curve (TD error over episode).

The temporal-difference (TD) loss curve in Figure 5 shows minimal volatility, as expected during DQN training because of replay-based updates, demonstrating consistent learning.

#### 4.2.3 Epsilon decay

**Figure 6:**
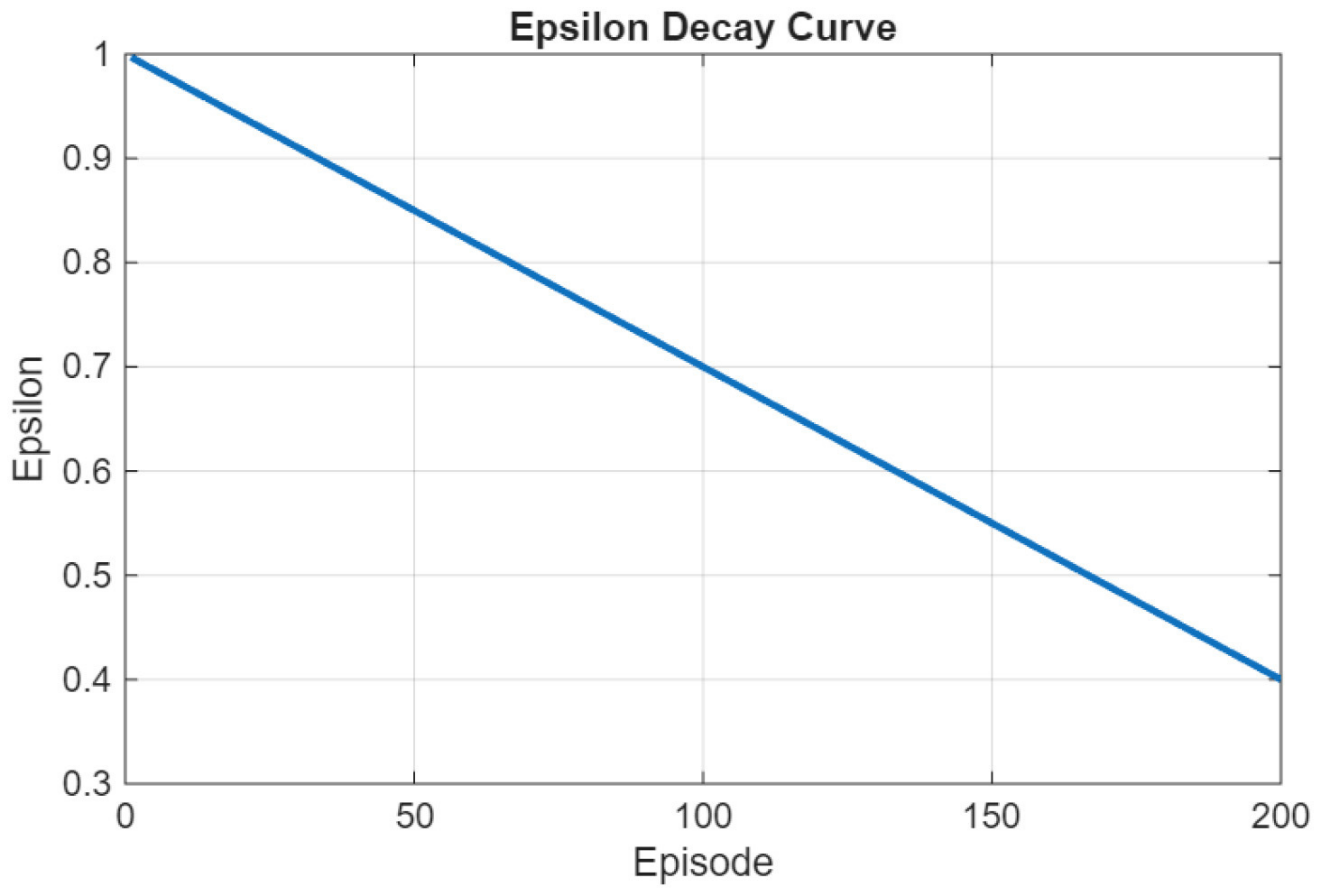
Epsilon decay curve.

Figure 6 shows the gradual decrease in the exploration rate ϵ during DQN training. Higher ϵ values at the beginning encourage experimentation with encryption activities, whereas lower ϵ values later on allow for the application of the learned optimal policy, resulting in consistent and predictable encryption behavior.

#### 4.2.4 Max Q-value evolution

**Figure 7:**
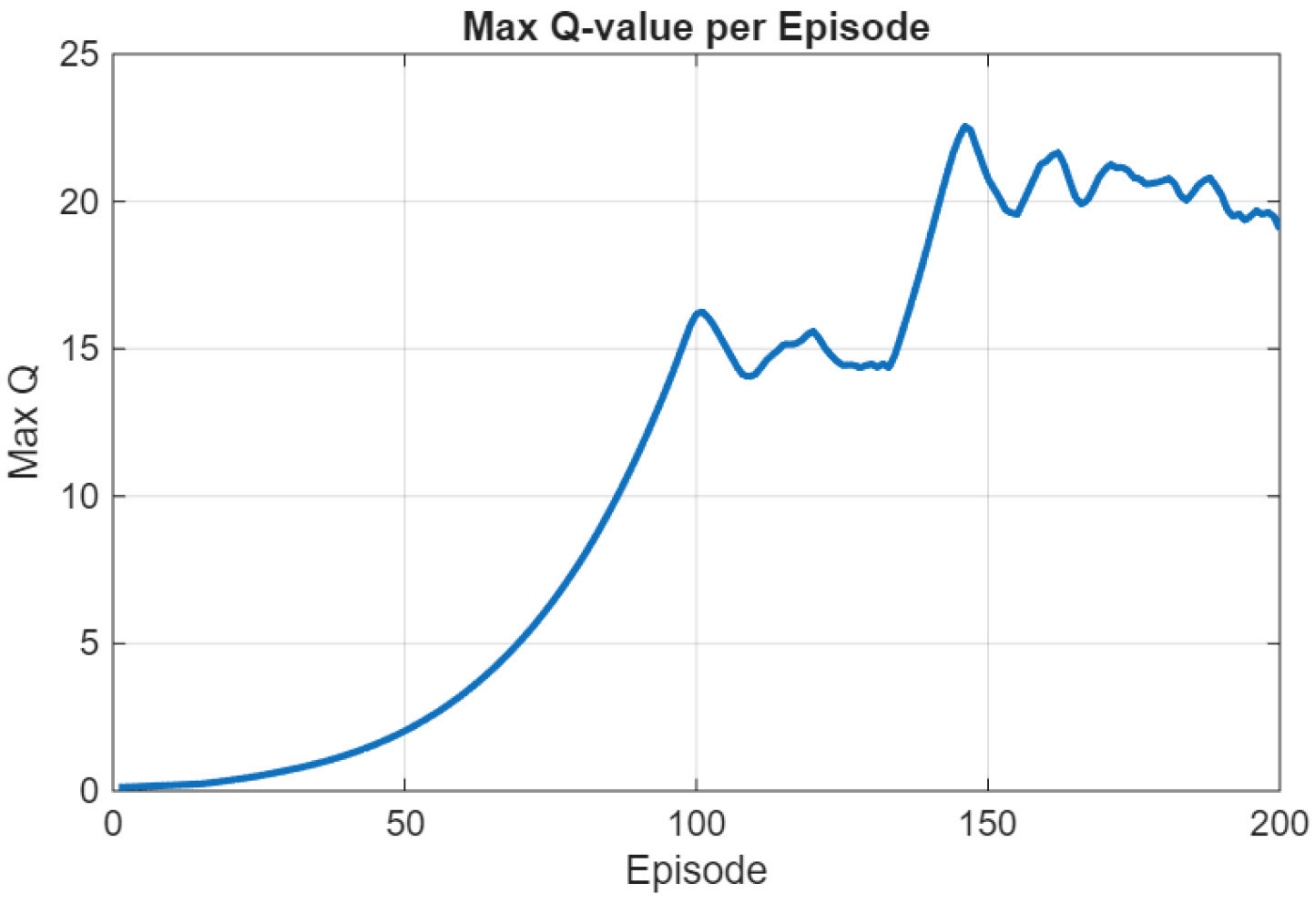
Max Q-value curve.

Figure 7 shows how the maximum Q-value changes across training episodes. The increasing rate implies that the DQN is gradually learning to encrypt activities that result in larger rewards, displaying excellent policy optimization and reinforcement learning process convergence.

#### 4.2.5 NPCR, UACI, episode reward curve and SSIM during training

**Figure 8:**
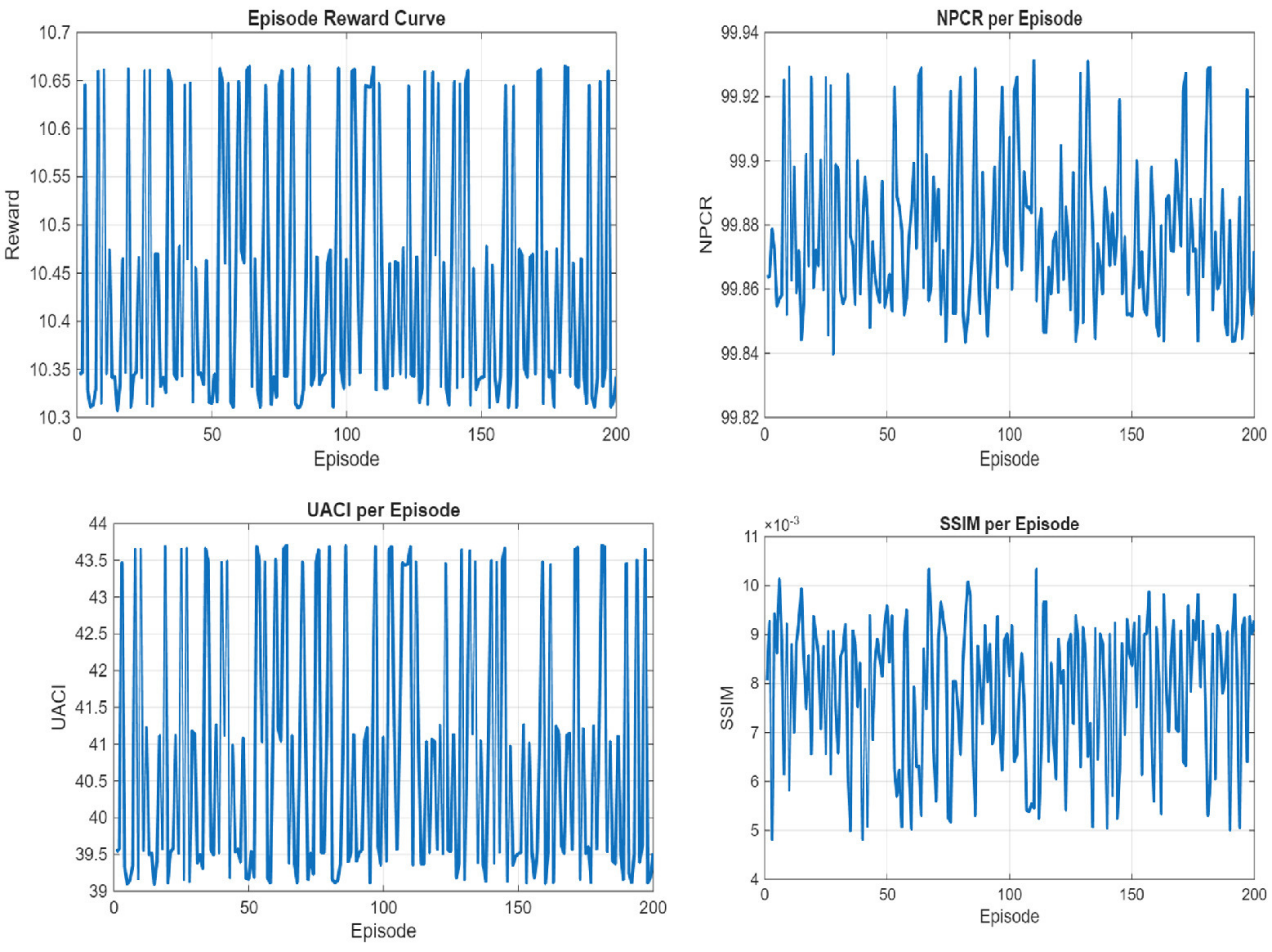
Reward curve, NPCR, UACI, SSIM.

The image shows the NPCR values across training episodes, illustrating that high pixel sensitivity is maintained throughout the policy-learning process. Figure 8 shows the effective optimization of the proposed cryptographic reward function, which includes episode, NPCR, UACI, and SSIM.

In summary, the results indicate that the DQN generates a stable and deterministic policy, which is critical for providing reproducible and fully reversible medical image encryption.

### 4.3 Security performance evaluation

#### 4.3.1 Histogram analysis

**Figure 9:**
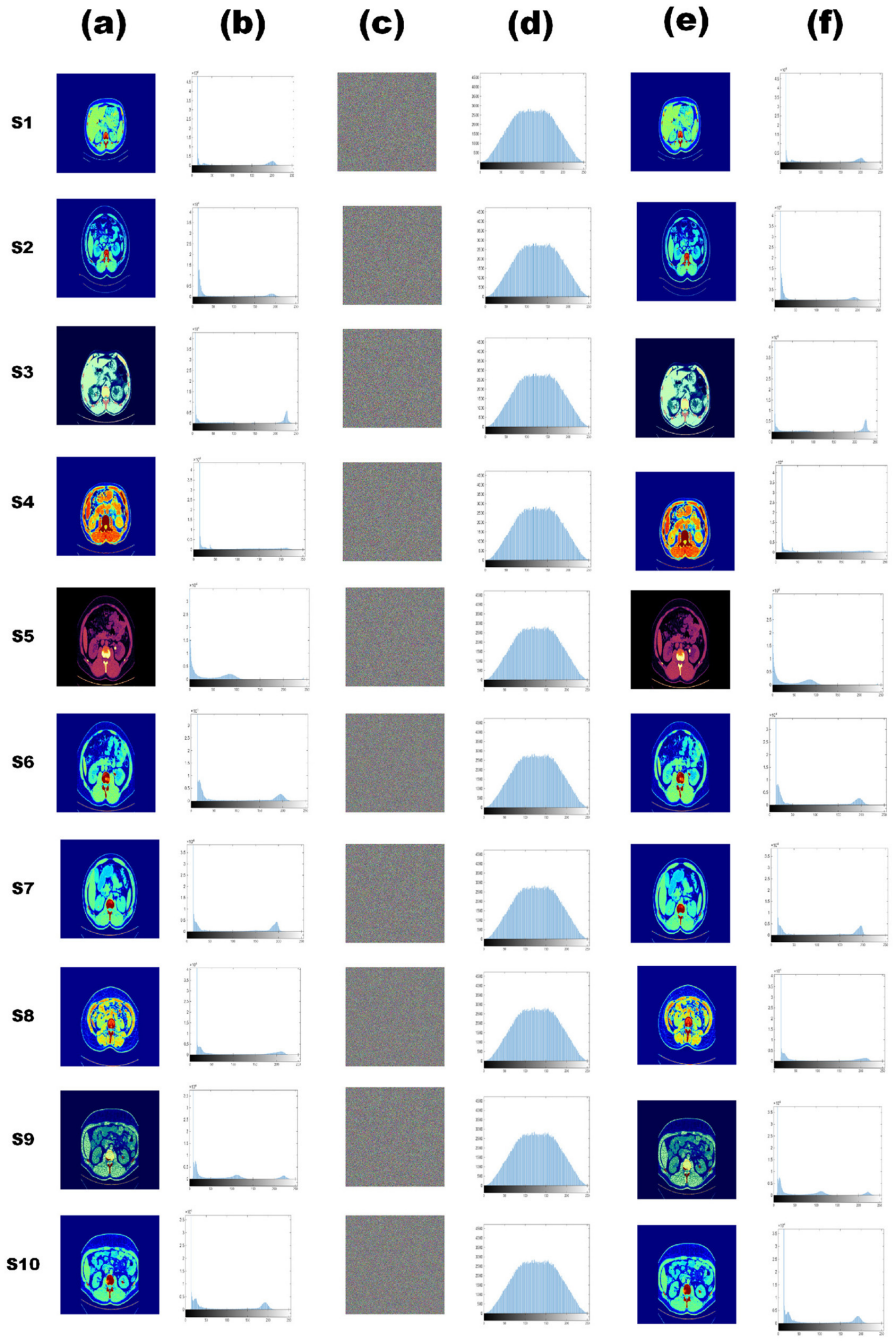
**(a)** Original medical images. **(b)** Histograms of original images. **(c)** Encrypted images. **(d)** Histograms of encrypted images. **(e)** Decrypted images. **(f)** Histograms of decrypted images.

A histogram displays the distribution of pixel intensities in an image. In a typical image, the histogram exhibits peaks that indicate the color. Encrypted images, in contrast, have histograms that appear more uniform and have fewer visible peaks because encryption seeks to erase patterns and make it more difficult to identify the original information (Ningthoukhongjam et al., 2024). Figure 9 shows the pixel distributions of the original and encrypted images.

#### 4.3.2 Differential attack analysis

A differential attack examines the sensitivity of an encryption algorithm to tiny variations in secret keys or plaintext. A secure encryption technique should produce noticeable changes in the ciphertext even if only one pixel of the plaintext is changed. The unified average changing intensity (UACI) and number of pixels change rate (NPCR) can be used to assess the resistance of an encryption method is against differential attacks. These metrics can be expressed mathematically as follows (Zhang et al., 2022):

The number of pixel change rate (NPCR) and unified average changing intensity (UACI) are widely used metrics to evaluate the resistance of an encryption scheme against differential attacks. These metrics measure the impact of a slight change in the plaintext image on the encrypted image and are calculated using Equations 2–4.


$$\begin{array}{c} NPCR=\left(\frac{\sum_{i=1}^{w}\sum_{j=1}^{h}D\left(i,j\right)}{w\times h}\right)\times 100\% \end{array}$$ (2)

$$\begin{array}{c} UACI=\left[\frac{1}{w\times h}\overset{w}{\underset{i=1}{\sum }}\overset{h}{\underset{j=1}{\sum }}\frac{\left|C_{1}\left(i,j\right)-C_{2}\left(i,j\right)\right|}{255}\right]\times 100\% \end{array}$$ (3)

$$\begin{array}{c} D\left(i,j\right)=\{\begin{array}{cc} 0, & \text{if }C_{1}\left(i,j\right)=C_{2}\left(i,j\right)\\ 1, & \text{if }C_{1}\left(i,j\right)\neq C_{2}\left(i,j\right) \end{array} \end{array}$$ (4)

The width and height of the original image *C* are denoted by *w* and *h*, respectively, while the first and second encrypted images are represented by *C*_1_ and *C*_2_. Table 3 lists the obtained NPCR and UACI results.

**Table 3:** NPCR and UACI values for the encrypted images.

Sample image	NPCR	UACI
S1	99.897%	37.18%
S2	99.898%	39.79%
S3	99.929%	33.12%
S4	99.854%	36.49%
S5	99.872%	43.94%
S6	99.87%	37.18%
S7	99.852%	39.79%
S8	99.859%	33.12%
S9	99.877%	36.49%
S10	99.851%	43.94%

Table 3 present that the NPCR and UACI values of the encrypted images closely approach the expected theoretical values. An NPCR value approaching 99.6% indicates strong diffusion characteristics. UACI values may exceed the theoretical grayscale benchmark (33.4635%) in practical encryption systems due to strong diffusion mechanisms that amplify pixel intensity variations and propagate changes across the image.

**Figure 10:**
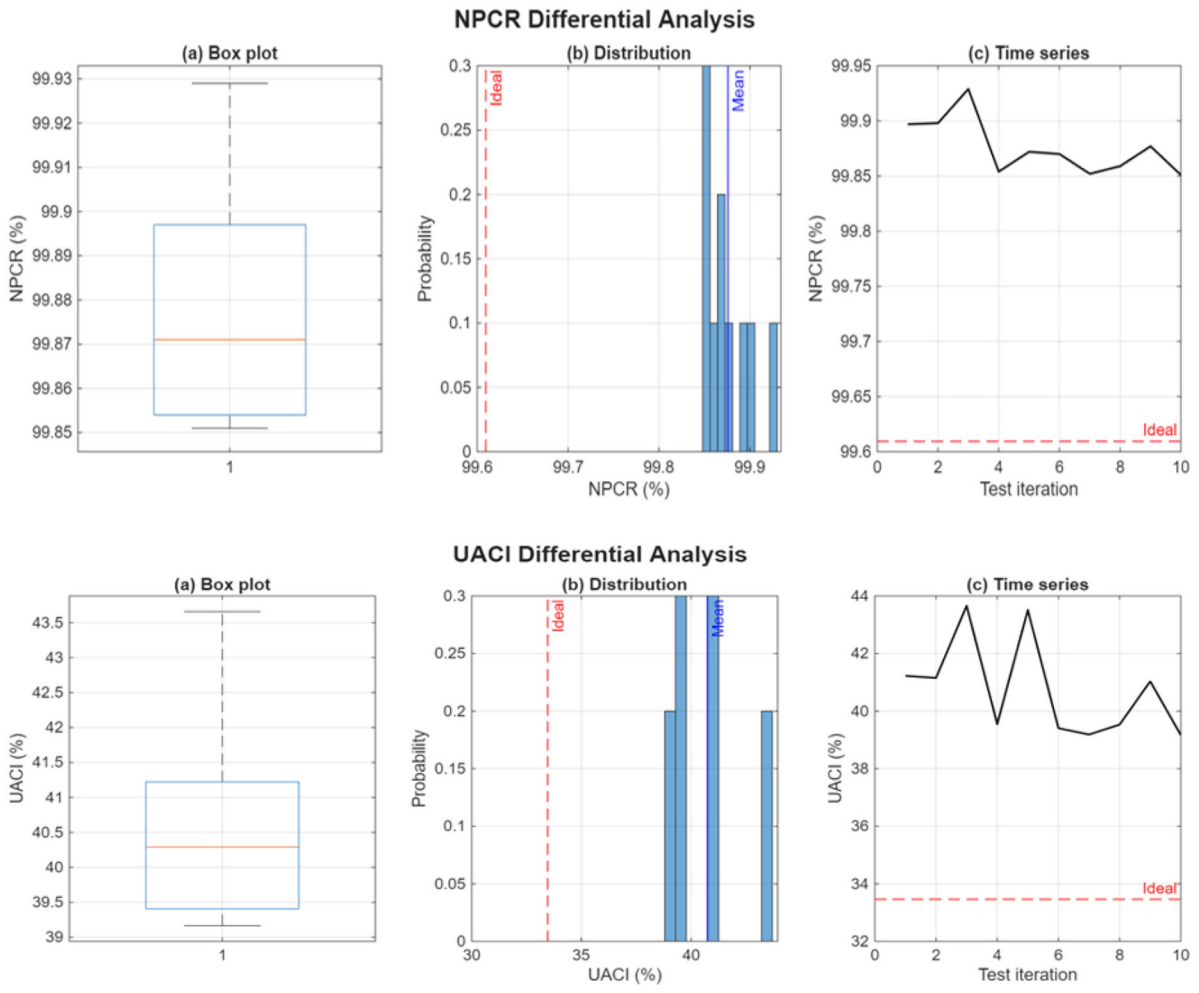
NPCR and UACI differential analysis. For NPCR: **(a)** box plot, **(b)** distribution, and **(c)** time series. For UACI: **(a)** box plot, **(b)** distribution, and **(c)** time series.

The experimental results demonstrate that the NPCR values exceed 99.8% whereas the UACI values remain within the normal range for RGB images, typically between 39% and 43%. Furthermore, the NPCR and UACI distribution analysis presented in Figure 10, uses box plots and histograms to illustrate stable clustering behavior consistent with theoretical expectations. The combined protection of independent channel diffusion and cross-channel propagation in RGB encryption leads to enhanced UACI values, thereby strengthening resistance against differential attacks (Hu et al., 2025). It is important to note that the theoretical UACI value of 33.4635% is derived for grayscale images under ideal random distribution assumptions. In this work, UACI is evaluated over RGB images by aggregating differences across all three channels. Due to multi-channel diffusion and feedback propagation, the resulting UACI values may exceed the grayscale benchmark. When computed on a per-channel basis, the UACI values remain close to theoretical expectations, thereby confirming the correctness of the computation.

#### 4.3.3 Entropy analysis

The degree of uncertainty in an image's information content can be evaluated using the entropy measurement. The entropy is computed using Equation 5 (Prabhavathi and Anandaraju, 2023).


$$\begin{array}{c} H=-\overset{256}{\underset{i=1}{\sum }}P\left(e_{i}\right)\log_{2}\left(\frac{1}{P\left(e_{i}\right)}\right) \end{array}$$ (5)

P(*e*_*i*_) represents the probability of (*e*_*i*_). The entropy value of a completely random image is close to 8. When the entropy value of the system decreases below, it becomes more predictable. the entropy values are listed in Table 4.

**Table 4:** Entropy analysis of test images.

Sample image	Entropy
S1	7.9995
S2	7.9996
S3	7.9996
S4	7.9996
S5	7.9996
S6	7.9996
S7	7.9996
S8	7.9996
S9	7.9996
S10	7.9995

#### 4.3.4 Key space analysis

The SHA-512-based seed derivation process is the primary component influencing the cryptographic strength of the proposed framework. Our proposed encryption scheme employs SHA-512, which generates 512-bit outputs to establish an effective key length of 512 bits. These data indicate that the theoretical key space is larger than 2^512^. To prevent the leakage of deterministic patterns, the system generates seeds using nonces. When nonce randomness is combined with current security methods, the effective key space increases beyond 2^512^, improving defense against exhaustive search and brute-force attacks. The system creates all chaotic parameters and Arnold scrambling rounds using deterministic methods that employ the seed to provide practical key diversity and full reversibility.

#### 4.3.5 Correlation analysis

The relationship between adjacent pixels is a key statistic for determining the effectiveness of a cryptosystem. The correlation coefficient between two adjacent pixels is calculated using Equation 6, where the covariance, variance, and expectation are defined in Equations 7–9, respectively.


$$\begin{array}{c} \text{corr}\left(x_{1},x_{2}\right)=\frac{\text{cov}\left(x_{1},x_{2}\right)}{\sqrt{D\left(x_{1}\right)}\times \sqrt{D\left(x_{2}\right)}} \end{array}$$ (6)

where the covariance between two adjacent pixels is given by


$$\begin{array}{c} \text{cov}\left(x_{1},x_{2}\right)=\frac{1}{n}\overset{n}{\underset{i=1}{\sum }}\left[\left(x_{i}-E\left(x_{1}\right)\right)\left(y_{i}-E\left(x_{2}\right)\right)\right] \end{array}$$ (7)

The variance is computed as


$$\begin{array}{c} D\left(x_{1}\right)=\frac{1}{n}\overset{n}{\underset{i=1}{\sum }}{\left(x_{i}-E\left(x_{1}\right)\right)}^{2} \end{array}$$ (8)

and the expectation (mean) is defined as


$$\begin{array}{c} E\left(x_{1}\right)=\frac{1}{n}\overset{n}{\underset{i=1}{\sum }}x_{i} \end{array}$$ (9)

**Figure 11:**
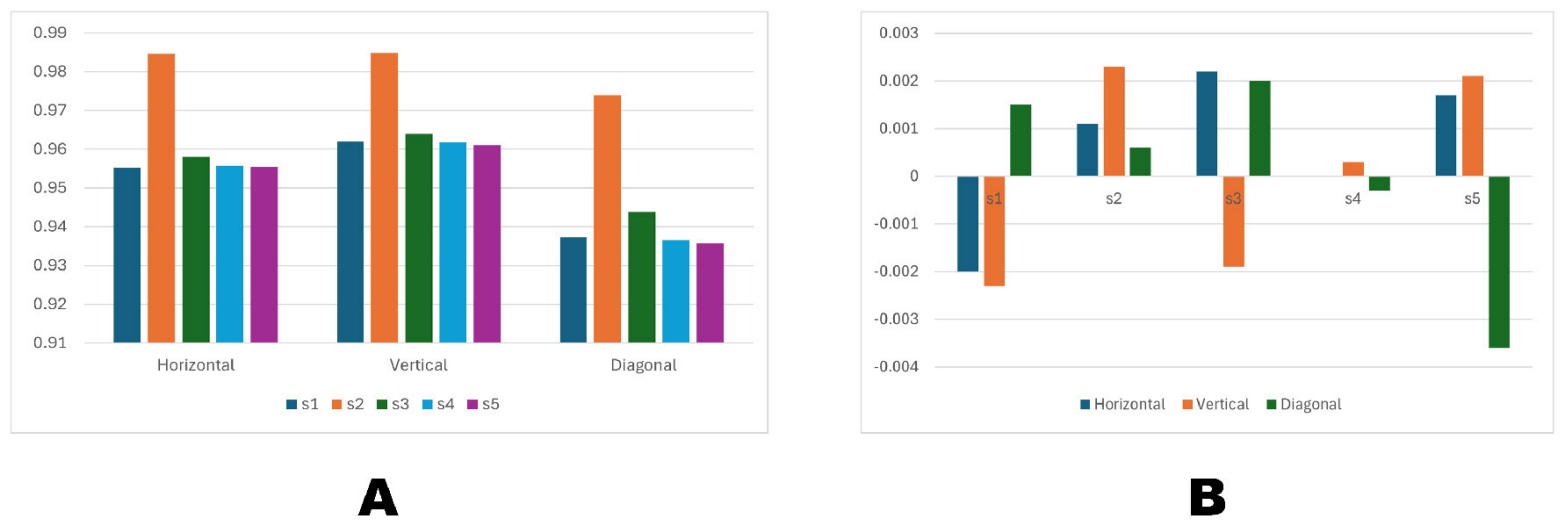
Correlation coefficient analysis: **(A)** Correlation coefficients of the original image and **(B)** Correlation coefficients of the encrypted image.

The values of the two adjacent pixels are *x*_1_ and *x*_2_, n is the total number of pixels, and *E*(*x*_1_), *D*(*x*_1_) are the expectation (mean) and variance, respectively. The differences in the correlation coefficients between the original and encrypted images are shown in Figure 11.

#### 4.3.6 Reconstruction accuracy analysis (MSE and PSNR)

The evaluation of encryption methods requires two essential metrics, namely, mean squared error (MSE) and peak signal-to-noise ratio (PSNR), which compare the original images with their encrypted versions. The MSE and PSNR values are computed using Equations 10 and 11, respectively


$$\begin{array}{c} MSE=\frac{\sum_{i=1}^{M}\sum_{j=1}^{N}{\left(I_{\left(i,j\right)}-EI_{\left(i,j\right)}\right)}^{2}}{M\times N} \end{array}$$ (10)

$$\begin{array}{c} PSNR=10\log_{10}\left(\frac{I_{\text{max}}^{2}}{MSE}\right) \end{array}$$ (11)

The image dimensions are represented as *M* × *N*, where *I*_(*i, j*)_ signifies the pixel value of the original image and *EI*_(*i, j*)_ denotes the pixel value of the accurately decrypted image. To validate the precise reversibility between the original and correctly decrypted images, mean squared error (MSE) and peak signal-to-noise Ratio (PSNR) calculations are utilized. For decrypted images, the reconstruction error is zero, resulting in MSE = 0 and PSNR = (∞ dB), which confirms exact pixel-level recovery. The values presented in Table 5 correspond to the comparison between the original and encrypted images, demonstrating significant distortion after encryption (Umar et al., 2024).

**Table 5:** MSE and PSNR values for encrypted images.

Image	MSE	PSNR (dB)
S1	15767.4227	6.15319649
S2	15890.6257	6.11939363
S3	16333.7804	5.99993650
S4	15979.3689	6.09520739
S5	18150.0863	5.54201667
S6	15702.8649	6.17101467
S7	15658.4428	6.18331791
S8	15978.5973	6.09541710
S9	15390.5895	6.25825106
S10	15871.7604	6.12455263

#### 4.3.7 SSIM analysis

The structural similarity index measure (SSIM) determines the encrypted image is to the original. Brightness, contrast, and structural aspects are its three primary bases. It is quantified between 0 and 1. The similarity between the original and encrypted images (El-Shafai et al., 2021) was suggested to increase as the SSIM increases. The SSIM is expressed as follows:

The structural similarity index measure (SSIM) was used to evaluate the structural similarity between two images. It measures changes in luminance, contrast, and structural information between the original and the encrypted images. The SSIM is calculated using Equation 12.


$$\begin{array}{c} SSIM\left(x,y\right)=\frac{\left(2\mu_{x}\mu_{y}+C_{1}\right)\left(2\sigma_{xy}+C_{2}\right)}{\left(\mu_{x}^{2}+\mu_{y}^{2}+C_{1}\right)\left(\sigma_{x}^{2}+\sigma_{y}^{2}+C_{2}\right)} \end{array}$$ (12)

where:

- μ_*x*_ is the average of image *x*, and μ_*y*_ is the average of image *y*.
- $\sigma_{x}^{2}$ and $\sigma_{y}^{2}$ represent the variance of images *x* and *y*, respectively.
- σ_*xy*_ denotes the covariance between *x* and *y*.
- $C_{1}={\left(K_{1}L\right)}^{2}$ and $C_{2}={\left(K_{2}L\right)}^{2}$ are small constants used to stabilize the division.

Table 6 lists the SSIM values for the original and encrypted images. The obtained SSIM values are close to zero when using the small constants *K*_1_ = 0.01 and *K*_2_ = 0.03, indicating very low structural similarity between the original and encrypted images. In contrast, the SSIM between the original and decrypted images is equal to 1, confirming perfect structural similarity and lossless reconstruction. These results demonstrate that the proposed encryption framework produces significant perceptual distortion in the encrypted images while ensuring complete reversibility during decryption. Therefore, the proposed image encryption model is highly effective and exhibits strong resistance against statistical attacks.

**Table 6:** SSIM values for encrypted images.

Sample image	SSIM
S1	0.002888693
S2	0.00325349
S3	0.003171423
S4	0.003250816
S5	0.002325005
S6	0.00350046
S7	0.003569053
S8	0.002965863
S9	0.002607527
S10	0.00281092

### 4.4 Ablation study

To validate the effectiveness of the proposed DQN-based adaptive action selection strategy, an ablation test is performed in which the RL technique was substituted with a fixed transformation approach whereas the rest of the cryptographic modules remained unchanged. The comparative results are summarized in Table 7.

**Table 7:** Ablation study evaluating the impact of the reinforcement learning module on encryption performance.

Model	Entropy	NPCR (%)	UACI (%)
Without RL	7.6322	99.6787	34.2245
Proposed (With RL)	7.9996	99.8759	40.7409

As shown in Table 7, the proposed reinforcement learning strategy considerably enhances the statistical unpredictability and strength of image diffusion, with the entropy value approaching a maximum value of 8 and the NPCR and UACI values increasing appropriately.

### 4.5 Robustness analysis

#### 4.5.1 Noise and data loss attacks

**Figure 12:**
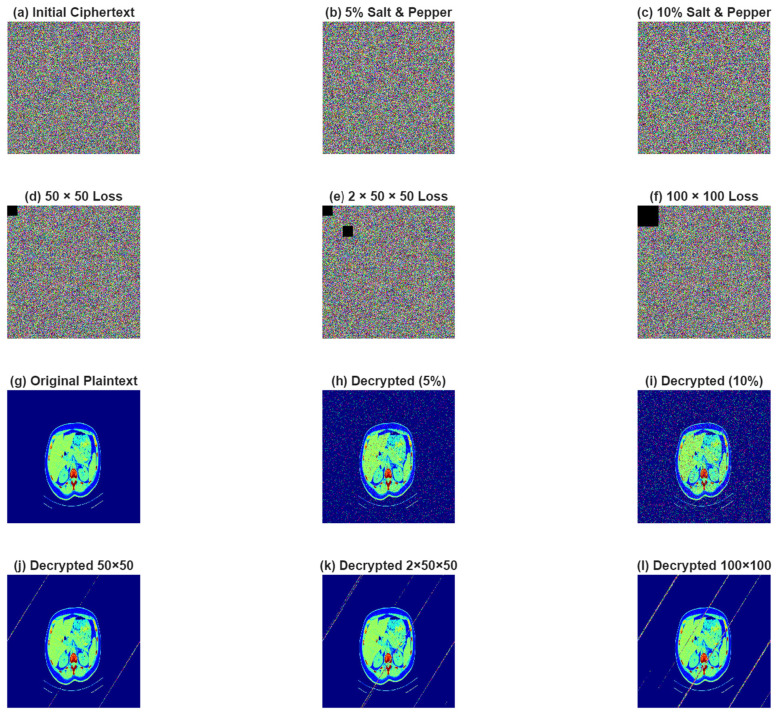
Results of noise and data loss attacks: **(a)** the initial ciphertext image, **(b)** 5% salt and pepper noise, **(c)** 10% salt and pepper noise, **(d)** 50 × 50 data losses, **(e)** two instances of 50 × 50 data losses, **(f)** 100 × 100 data losses, **(g)** the original plaintext image, **(h–l)** the decrypted images corresponding to **(b–f)**, respectively.

It is essential to employ a robust algorithm for encrypted images to ensure inaccessibility in the event of data loss during transmission or storage, as well as the capacity to decrypt lost data blocks or pixels (Zhang et al., 2024). It must also be robust to noise attacks, such as salt and pepper, Gaussian, or Poisson noise, which may interfere with image recovery (Eltoukhy et al., 2025). The performance of the algorithm was also evaluated under such circumstances. Figure 12 illustrates five methods of attacks on the ciphertext: (1) 5%, (2) 10%, (3) 50 × 50, (4) two 50 × 50, and (5) 100 × 100. During the attack, the ciphertext was decrypted using the appropriate key, and the PSNR and SSIM values for both the original and decrypted images were computed (Zhang et al., 2021). Table 8 lists the results of the attacks, with high values indicating strong similarities between the images.

**Table 8:** Evaluation of performance under noise and data loss attacks.

Noise type/loss type	Level	PSNR	SSIM
5% SP noise	0.05	19.223	0.8962
10% SP noise	0.10	16.203	0.8035
50 × 50 loss	1	28.128	0.9789
2 × 50 × 50 loss	2	25.175	0.95825
100 × 100 loss	3	22.292	0.91945

#### 4.5.2 Occlusion attack

**Figure 13:**
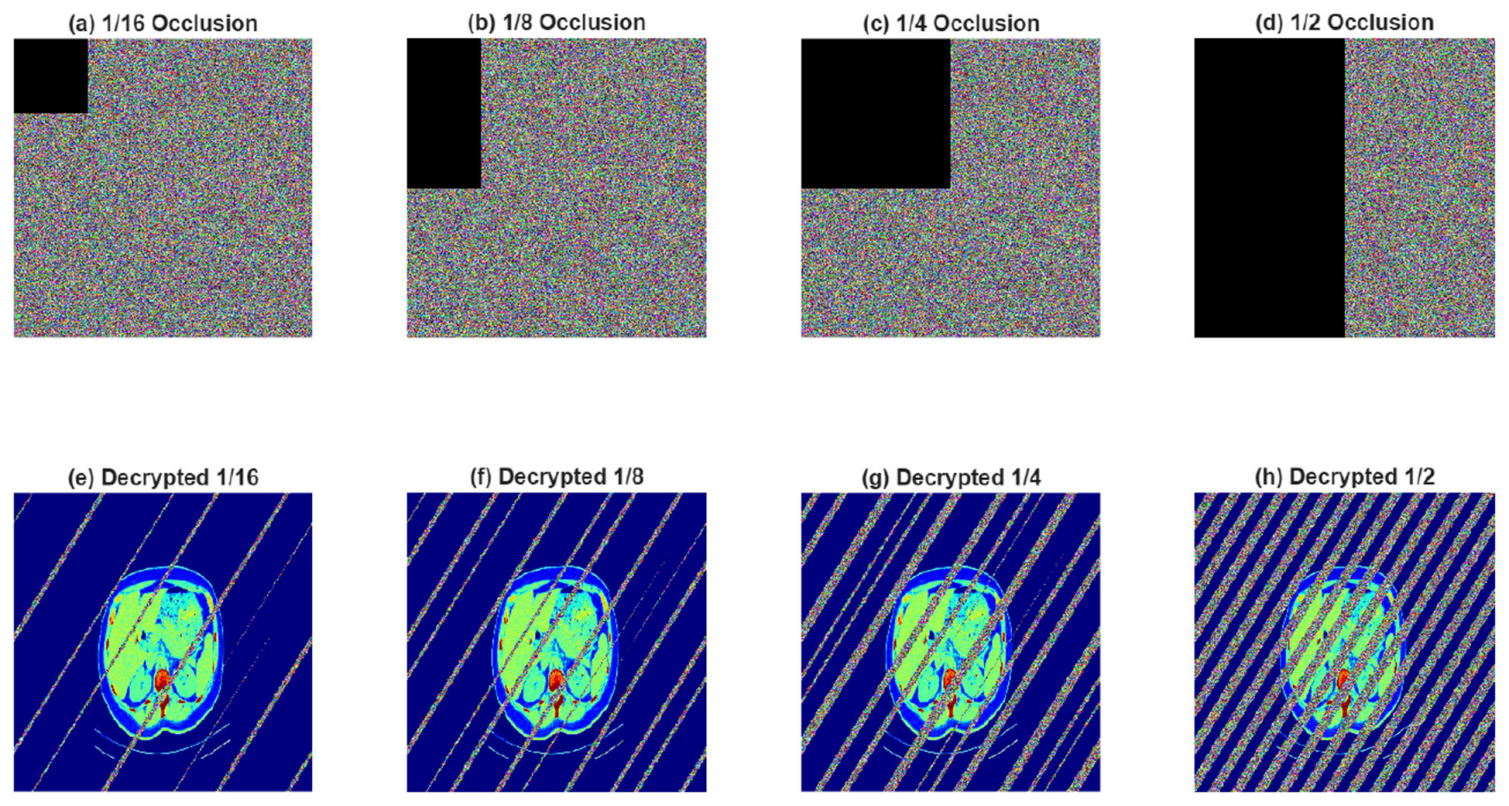
Image occlusion attacks were tested using encrypted images with data loss of **(a)** 1/16, **(b)** 1/8, **(c)** 1/4, and **(d)** 1/2, along with decrypted images **(e–h)** corresponding to **(a–d)**.

Encrypted images may lose data during transmission, which can affect the decryption process. An occlusion attack on encrypted images with varying levels of data loss (1/16, 1/8, 1/4, and 1/2) was conducted to evaluate the robustness of the algorithm. The visual results of the occlusion attack analysis are presented in Figure 13. The results of the encrypted images demonstrated that the approach retained significant visual information even when up to 50% of the data was destroyed, indicating its resilience (Zhang et al., 2021). In addition, the PSNR values for the proposed algorithms are presented in Table 9.

**Table 9:** PSNR values under different occlusion attack ratios.

Metric	1/16	1/8	1/4	1/2
PSNR (dB)	18.218	15.194	12.192	9.1797

#### 4.5.3 Chosen plaintext attack

**Figure 14:**
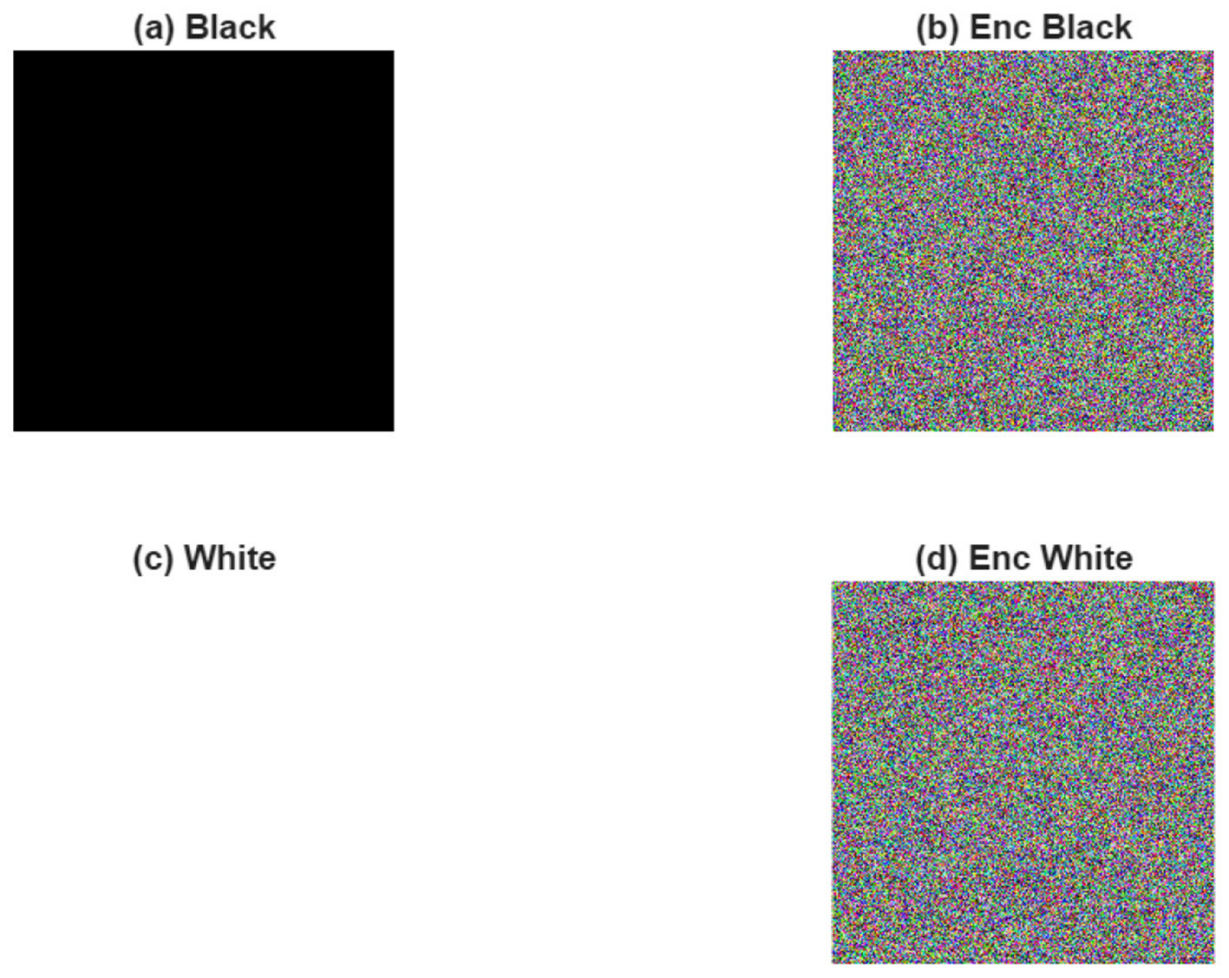
Chosen plaintext attack **(a)** Black image, **(b)** Encrypted image of Black image, **(c)** White image, and **(d)** Encrypted image of White image.

In a chosen plaintext attack, the attacker can encrypt a selected image, Such as a black image Figure 14a, to determine the secret key used. The attacker has temporary access to the encryption algorithm. The attacker obtains the chaotic data utilized in the encryption process (the secret key) after encrypting this black image Figure 14b. The strength of the proposed encryption against specific plaintext attacks was confirmed by repeating this process with a white image Figures 14c, d (Banga et al., 2025).

NPCR and UACI were assessed by comparing encrypted copies of fully black and white images. The NPCR and UACI values obtained were 99.62%, while the UACI value is 33.51%, which are close to their optimal levels. Table 10 shows that considerable changes in the plaintext result in significant alterations in the ciphertext, providing effective protection against chosen plaintext attacks.

**Table 10:** Information entropy and correlation coefficients of encrypted images.

Image	Information entropy	Correlation (horizontal)	Correlation (vertical)	Correlation (diagonal)
All black	7.9998	0.00270	–0.00091	–0.00067
All white	7.9998	0.00261	–0.00194	–0.00181

#### 4.5.4 Key sensitivity analysis

**Figure 15:**
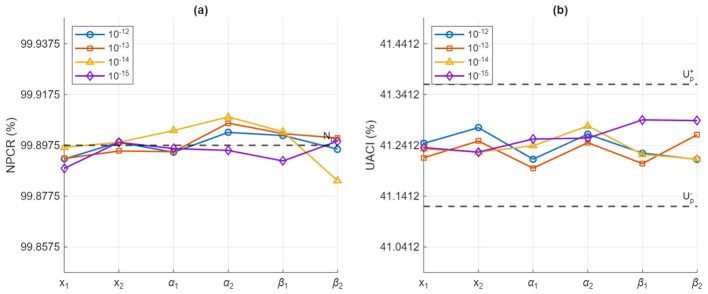
Key sensitivity analysis: **(a)** NPCR values under different parameter perturbations and **(b)** UACI values under different parameter perturbations.

Small modifications were applied to each part of the secret key for further statistical analysis, thereby obtaining two encrypted versions images for the NPCR and UACI calculations. As shown in the Figure 15, the NPCR and UACI values are within the critical region, indicating that the proposed encryption method is highly sensitive to even small key changes (Mansouri et al., 2025). This also strengthens the proposed system's resistance to cryptanalysis of the key.

#### 4.5.5 Determinism leakage test

During encryption of the same image using the same secret key and nonce, identical ciphertexts are observed, which is expected in a deterministic cryptographic system. To prevent equality inference attacks, a nonce-based seed derivation mechanism is introduced. When different nonces are used with the same secret key, the ciphertext difference was found to be greater than 99.62%.

### 4.6 RL policy robustness

#### 4.6.1 DQN policy stability test

**Figure 16:**
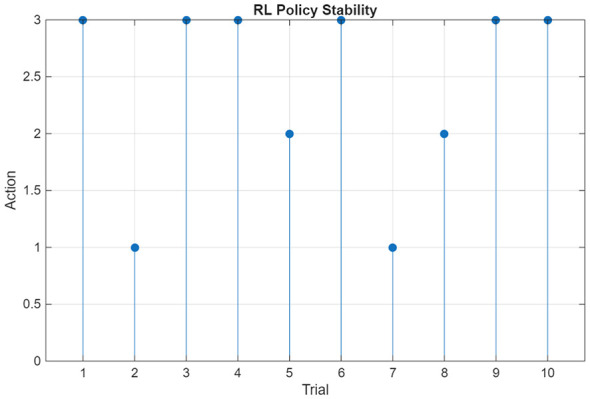
RL policy stability.

The policy stability test evaluates the durability of the learned reinforcement learning model by continually providing it a fixed feature state learned throughout the reinforcement learning process as input to the learned deep Q-network (DQN). The model's learned action sequence, shown in Figure 16, shows slight differences between Actions 1, 2, and 3, however Action 3 continues to dominate most trials. As shown in Figure 16, the learned model consistently chooses Action 3 owing to deterministic greedy selection, suggesting that the reinforcement learning model is steadily converging toward a dominating optimal policy based on the learned feature state. Despite the learned model's deterministic qualities, the the security of encryption process is unaffected because cryptographic randomness is guaranteed by various approaches, including nonce-based seed generation, SHA-512 masking, Arnold scrambling, and chaos theory-based diffusion.

#### 4.6.2 Adversarial feature perturbation

The adversarial feature perturbation test evaluates the robustness of a policy by integrating random perturbations into the feature vector. Here, Action 3 is consistently chosen by the agent in every trial. The results demonstrate strong decision boundary stability, demonstrating that the learned policy is unaffected by modest changes in feature inputs. The robustness of the learned policy is desirable in cryptographic systems because it ensures that the policy runs consistently even with modest changes in feature extraction. The convergence of the learnt policy is further demonstrated by the stability of action selection.

#### 4.6.3 Action prediction test

**Figure 17:**
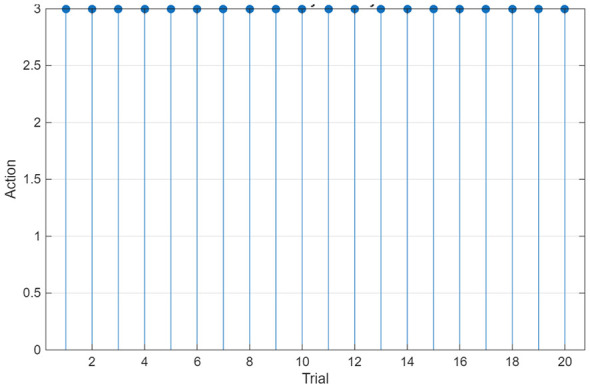
Action prediction test.

The action prediction evaluation for the action prediction evaluation assesses the confidence of the trained DQN through the evaluation of Q-value dominance for a fixed feature state. As shown in Figure 17, Action 3 has the highest Q-value in all evaluations, far surpassing the other actions. The gap between the Q-values indicates that the policy has been learned with great confidence and accuracy in decision-making. Although the policy is deterministic in its decision-making, as shown in the action prediction evaluation, the randomness of the encryption process is maintained through independent processes of chaos-based diffusion and random key generation using nonces. Thus, the determinism of the action prediction does not affect the security of the encryption process.

#### 4.6.4 Action sequence diversity

**Figure 18:**
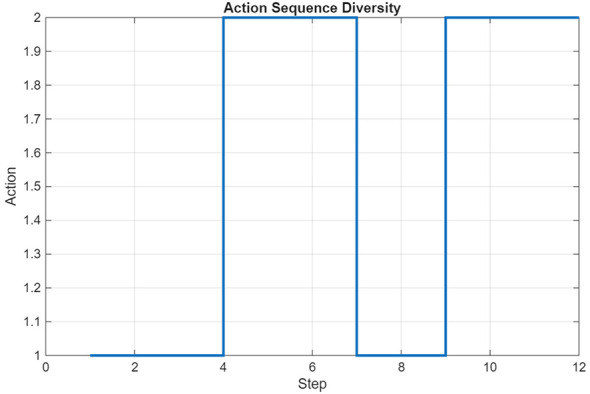
Action sequence diversity.

The action sequence diversity experiment investigates the dynamic reaction of a reinforcement learning agent to an encryption method. In contrast to the policy stability test, this experiment tests the reaction of the reinforcement learning agent to determine whether it dynamically modifies the chosen action based on the state change in the image during the iterative encryption process. Figure 18 shows the action sequence. The results indicate, that Action 3 is the with action in most cases; however, Actions 1 and 2 can also be employed in other scenarios. This demonstrates that the policy has already converged to a certain course of action that maximizes the reward metrics and, thus, guarantees image security. Although the action sequence is relatively diverse, the randomness of the process is ensured by various individual techniques, including SHA-512, masking, Arnold scrambling, seed generation, and chaos, which are employed in image encryption. Thus, the dominance of a single action has no impact on the security of the proposed framework but is a result of the policy's convergence to a stable state according to the optimization function.

### 4.7 Computational complexity analysis

The computational complexity of the proposed encryption algorithm is O(H × W × T), where T is the number of encryption steps and H and W represent the image height and width, respectively. Since the number of encryption steps T remains relatively small and constant, the overall computational complexity scales approximately linearly with the image size, making the proposed framework suitable for high-resolution medical imaging applications. Each encryption stage comprises pixel-level processes such as keystream masking, chaotic diffusion, and Arnold scrambling. Because T is modest and constant, the total complexity is virtually linear with respect to the image size. The decryption process follows the same number of reversible steps; therefore, its computational complexity is also O(H × W × T). This linear scalability makes the proposed framework suitable for high-resolution medical image encryption in IoMT environments.

### 4.8 Computational efficiency

The computational efficiency of the proposed reversible DQN-based encryption method was assessed by determining the execution time per image. While decryption takes approximately 0.9 s per image, the average encryption duration is 1.08 s. The combination of SHA-512 masking, chaotic diffusion, Arnold scrambling, and transformation selection based on reinforcement learning makes the encryption process comparatively more time-consuming. In contrast, decryption requires predetermined inverse processes, resulting in a marginally shorter execution time. A stable computing efficiency is indicated by the low variance in execution durations across several test images. The proposed framework requires more execution time than lightweight chaos-only encryption schemes. This advantage results from the system's use of an adaptive reinforcement learning control, which also delivers improved security through enhanced diffusion protection and maintains strict reversibility requirements and prevents all types of determiniatic leakage.

### 4.9 IoMT performance analysis

To evaluate the suitability of the proposed framework for IoMT environments, key performance aspects such as latency, computational overhead, energy consumption, throughput, and real-time feasibility are analyzed.

#### 4.9.1 Latency, throughput, and execution time

The proposed reinforcement learning-based reversible image encryption framework achieved an average encryption latency of 2.8548 s per image with an average throughput of 0.4111 MB/s. The decryption process also demonstrated stable execution behavior suitable for secure medical image recovery. These results indicate that the framework supports secure transmission and storage of medical images in telemedicine and cloud-assisted healthcare environments. Although the latency is higher than lightweight stream-based encryption methods, the framework provides enhanced security through multi-stage encryption, reversible transformation, chaotic diffusion, and adaptive DQN-guided encryption operations.

#### 4.9.2 Computational overhead

The computational complexity of the proposed framework is approximately *O*(*H* × *W* × *T*), where *H* and *W* denote image dimensions and *T* represents the number of encryption stages. The framework achieved an average computational overhead of approximately 65.47 MB during execution. The use of lightweight operations such as XOR masking, SHA-512-based masking, chaotic diffusion, and a compact DQN architecture with two hidden layers helps maintain moderate memory utilization while preserving strong security characteristics. The primary computational cost arises from iterative Arnold scrambling operations.

#### 4.9.3 Energy consumption considerations

The average estimated energy consumption of the proposed framework was approximately 128.4667 Joules per image during encryption execution. Although direct hardware-level power profiling was not performed, the proposed approach mainly relies on lightweight pixel-level transformations and compact neural inference rather than computationally intensive deep CNN architectures. Compared to deep learning-based encryption frameworks requiring large convolutional operations, the proposed method is expected to exhibit lower computational and energy overhead, making it suitable for edge-assisted IoMT healthcare environments. Detailed hardware-specific energy profiling is considered as future work.

#### 4.9.4 Real-time feasibility

The proposed framework is designed for edge-assisted IoMT deployment, where encryption operations are executed at gateway, fog, or edge nodes instead of highly resource-constrained medical sensors. The observed near-linear computational complexity, moderate memory usage, and stable execution characteristics demonstrate the applicability of the framework for near real-time healthcare security applications, including telemedicine image transmission, secure cloud storage, remote diagnostics, and medical forensic archival systems. While the current implementation prioritizes security robustness and reversibility over ultra-low latency, future optimization through vectorized scrambling and GPU acceleration can further improve real-time deployment feasibility.

### 4.10 Benchmark dataset validation (MedMNIST)

The MedMNIST dataset was used to assess the generalizability of the proposed DRL-based reversible medical image encryption framework through benchmark validation testing. The encryption technique requires the conversion of grayscale MedMNIST images into RGB format. The same experimental design and security metrics used in the primary dataset were applied to test five representative images. The results showed entropy values that approached the theoretical maximum, NPCR values that exceeded 99.9%, UACI values that indicated good diffusion, and SSIM values that approached zero. The results demonstrate that the encryption system maintains strong performance across different, which making it suitable for Internet of Medical Things applications that protect medical images. Table 11 presents the entropy, NPCR, UACI, and SSIM values obtained for the five representative MedMNIST images.

**Table 11:** Performance evaluation metrics for benchmark images.

Sample image	Entropy	NPCR (%)	UACI (%)	SSIM
M1	7.9994	99.902	44.649	0.0037148
M2	7.9992	99.900	45.733	0.009382
M3	7.9991	99.878	44.382	0.008064
M4	7.9992	99.976	46.184	3.2966 × 10^−5^
M5	7.9994	99.927	46.441	0.00026715

## 5 Discussion

The results demonstrate that deep reinforcement learning enhances the adaptability of reversible encryption systems while maintaining strong security and perfect reconstruction capabilities. The Deep Q-Network (DQN) generates diverse encryption strategies by analyzing statistical patterns of input images, enabling dynamic modification of diffusion and confusion operations. The use of nonce-based seed derivation combined with chaos-driven diffusion ensures unpredictable ciphertext generation, thereby protecting against attacks that rely on identical plaintext patterns. The proposed method achieves NPCR values comparable to existing approaches while demonstrating improved UACI, indicating enhanced diffusion performance along with strict reversibility. Unlike traditional methods that rely on fixed transformation sequences, the proposed framework adapts to varying image characteristics through dynamic action selection. The comparison results in Table 12 indicate that the proposed framework achieves higher NPCR and UACI values, along with competitive entropy, compared with several recent state-of-the-art image encryption methods. Furthermore, the integration of reinforcement learning with deterministic cryptographic transformations enables adaptive diffusion behavior while preserving computational feasibility, making the proposed method suitable for secure IoMT environments.

**Table 12:** Comparison of the proposed method with existing image encryption techniques.

References	Methods/year	NPCR (%)	UACI (%)	Entropy	Enc/Dec time
Zhang et al. (2026)	Hyperchaotic + finite field-based image encryption	99.6019	33.45	7.9992	–
Inam et al. (2024)	Deep Learning-based image encryption using Cycle GAN (2024)	99.6826	33.2778	7.2120	–
Long et al. (2024)	Deep Learning-based image encryption (2024)	99.52	33.38	7.9740	–
Selvakumar et al. (2024)	Hybrid image encryption + Steganography + chaos + DNA encoding (2024)	99.61	42.83	7.99	10 s / 12 s
Ningthoukhongjam et al. (2024)	Hybrid public key image encryption (ECC + Blum-Goldwasser + chaos) (2024)	99.6901	33.5260	7.9996	0.075 s/0.066 s
Hu et al. (2025)	3D hyperchaotic image encryption with multi-layer cascade architecture (2026)	99.6095	33.4617	7.9990	0.2530 s/0.2302 s
Umar et al. (2024)	Modified skew tent chaotic map-based image encryption (2024)	99.6019	33.4562	7.9974	–
**Proposed**	**Hybrid Deep Reinforcement Learning (DRL) + Cryptographic Image Encryption**	**99.927**	**43.658**	**7.9996**	**1.08 s/0.9 s**

Bold values indicate the best-performing results among the compared methods.

In addition to the above analyses, the statistical randomness of the encrypted output is evaluated using the NIST SP 800-22 test suite. The evaluation is conducted on 10 independent bitstreams, each of length 10^6^ bits. The results, summarized in Table 13, indicate that all major tests satisfy both the *p*-value (>0.01) and proportion criteria defined by the NIST standards. These findings confirm that the proposed encryption framework produces statistically random ciphertext and exhibits strong resistance against statistical attacks.

**Table 13:** NIST SP 800-22 statistical test results.

Test	*p*-value	Result
Frequency	0.0179	PASS
Block frequency	0.1223	PASS
Runs	0.3505	PASS
Rank	0.5341	PASS
FFT	0.5341	PASS
Universal	0.5341	PASS
Approximate entropy	0.3505	PASS
Serial (*p*_1_)	0.5341	PASS
Serial (*p*_2_)	0.2133	PASS
Linear complexity	0.5341	PASS

## 6 Conclusion

A reversible medical image encryption method that uses deep reinforcement learning for secure image transmission in Internet of Medical Things (IoMT) environments is proposed. The method establishes secure protection with full decryption capabilities through its combination of deep Q-network (DQN) technology and a multi-stage cryptographic system. The encryption process uses adaptive policy learning to modify encryption parameters according to different image characteristics, thereby providing a solution to the limitations of fixed-parameter methods. The system achieves enhanced security through the combination of nonce-based randomness and chaotic diffusion techniques which provides protection against both determinism exposure and chosen-plaintext attacks. The system demonstrates its resilience against common transmission issues, including noise, occlusion and partial data loss, thus proving its value for medical applications. Future research will focus on creating lightweight deep reinforcement learning models for ultra-low-power Internet-of-Things for medical treatment (IoMT) devices, as well as investigating hardware-aware optimizations for real-time deployment.

## 7 Limitation

Although the proposed framework performs well, it has certain limitations. First, employing reinforcement learning-managed transformations and multi-stage encryption adds computational complexity, resulting in a modest increase in encryption time when compared to lightweight chaos-only approaches, which may pose challenges for ultra-low-power Internet of Things (IoMT) devices. Second, the existing method processes images offline, thereby ignoring the need for real-time streaming. The reinforcement learning agent training uses a permanent reward system that depends on defined security standards; however, system updates are required when new threats or adversarial objectives emerge. Grayscale images are replicated across RGB channels to enable uniform processing in the proposed framework. However, this approach may not fully preserve the modality-specific characteristics inherent to certain medical imaging formats. Additionally, the current implementation does not explicitly address DICOM metadata protection or compression artifacts, which should be considered in future extensions.

## 8 Future work

Future research should focus on enhancing real-time encryption methods used in edge-based IoMT devices through model compression, lightweight reinforcement learning, and hardware acceleration techniques. The development of DICOM-compliant encryption requires a framework that provides complete support for native DICOM-based metadata protection. The implementation of multi-agent reinforcement learning will enable remote IoMT nodes to work together on security policy development. Further research could explore transformer-based and actor-critic methods for developing advanced adaptive encryption systems. The decision-making process will improve through explainable artificial intelligence (XAI) methods because these methods make systems easier to understand, resulting in higher trust and recognition within clinical environments.

## Data Availability

The original contributions presented in the study are included in the article/supplementary material, further inquiries can be directed to the corresponding author.

## References

[B1] Aashiq Banu S. Rao L. K. Priya P. S. Thanikaiselvan Hemalatha, M. Dhivya R. (2025). A review of genome to chaos: exploring DNA dynamics in security. Multimedia Tools Applic. 84, 24859–24886. doi: 10.1007/s11042-024-20074-5

[B2] Abdellatef E. Naeem E. A. El-Samie F. E. A. (2024). DeepEnc: deep learning-based CT image encryption approach. Multimedia Tools Applic. 83, 11147–11167. doi: 10.1007/s11042-023-15818-8

[B3] Almeida B. A. Doneda D. Ichihara M. Y. Barral-Netto M. Matta G. C. Rabello E. T. . (2020). Personal data usage and privacy considerations in the COVID-19 global pandemic. Ciencia Saude Coletiva 25, 2487–2492. doi: 10.1590/SciELOPreprints.511 32520293

[B4] Alqahtani F. Amoon M. El-Shafai W. (2022). A fractional Fourier based medical image authentication approach. Comput. Mater. Continua 70, 3133–3150. doi: 10.32604/cmc.2022.020454

[B5] Aminanto M. E. Kim K. (2016). “Detecting impersonation attack in WiFi networks using deep learning approach,” in Proceedings of the International Workshop on Information Security Applications (Cham: Springer), 136–147. doi: 10.1007/978-3-319-56549-1_12

[B6] Banga A. Abbas A. Ali D. Innab N. Alluhaidan A. S. Iqbal N. . (2025). A novel pythonic paradigm for image encryption. Sci. Rep. 15:17076. doi: 10.1038/s41598-025-89397-z 40379821 PMC12084594

[B7] Basak S. (2023). Kidney Stone Axial CT Imaging Colorized Dataset. Kaggle Dataset. Available online at: https://www.kaggle.com/datasets/shuvokumarbasakbd/kidney-stone-axial-ct-imaging-colorized-dataset (Accessed January 28, 2026).

[B8] Bashir Z. Malik M. G. A. Hussain M. Iqbal N. (2022). Multiple RGB images encryption algorithm based on elliptic curve, improved Diffie–Hellman protocol. Multimedia Tools Applic. 81, 3867–3897. doi: 10.1007/s11042-021-11687-1

[B9] Belazi A. Mabrouk B. (2025). A refined sine-derived chaotic map for securing medical image encryption in telemedicine. Comput. Biol. Med. 196:110667. doi: 10.1016/j.compbiomed.2025.110667 40644892

[B10] Benssalah M. Rhaskali Y. (2020). “A secure DICOM image encryption scheme based on ECC, linear cryptography and chaos,” in Proceedings of the International Conference on Communications, Control Systems and Signal Processing (IEEE), 131–136. doi: 10.1109/CCSSP49278.2020.9151462

[B11] Bhattacharjee S. Gupta M. Chatterjee B. (2023). Time efficient image encryption–decryption for visible and COVID-19 X-ray images using modified chaos-based logistic map. Appl. Biochem. Biotechnol. 195, 2395–2413. doi: 10.1007/s12010-022-04161-7 36152105 PMC9510176

[B12] Deb S. Biswas B. Bhuyan B. (2019). Secure image encryption scheme using high efficiency word-oriented feedback shift register over finite field. Multimedia Tools Applic. 78, 34901–34925. doi: 10.1007/s11042-019-08086-y

[B13] El-Damak D. Alexan W. Mamdouh E. El-Aasser M. Fathy A. Gabr M. (2024). Fibonacci q-matrix, hyperchaos, and Galois field 2^8^ for augmented medical image encryption. IEEE Access. 12, 102718–102744. doi: 10.1109/ACCESS.2024.3433499

[B14] El-Shafai W. Aly M. H. Algarni A. D. El-Samie F. E. A. Soliman N. F. (2022). Secure and robust optical multi-stage medical image cryptosystem. Comput. Mater. Continua 70, 895–913. doi: 10.32604/cmc.2022.018545

[B15] El-Shafai W. Khallaf F. El-Rabaie E. M. El-Samie F. E. A. (2021). Robust medical image encryption based on DNA-chaos cryptosystem. J. Ambient Intell. Human. Comput. 12, 9007–9035. doi: 10.1007/s12652-020-02597-5

[B16] Eltoukhy M. M. Alsubaei F. S. Elnabawy Y. M. Hosny K. M. (2025). Multiple image encryption techniques. Alexandria Eng. J. 125, 367–387. doi: 10.1016/j.aej.2025.04.006

[B17] He P. Huang D. Wu D. He H. Wei Y. Cui Y. . (2024). A survey of internet of medical things: technology, application and future directions. Dig. Commun. Netw. 12, 717–742 doi: 10.1016/j.dcan.2024.11.013

[B18] Hu C. Zhang L. Chen R. Gu Q. Ning B. Hua Q. . (2025). A dynamically parameterized 3D hyperchaotic cascade for secure encryption of medical images. Dig. Sig. Process. 170:105767. doi: 10.1016/j.dsp.2025.105767

[B19] Inam S. Kanwal S. Anwar A. Mirza N. F. Alfraihi H. (2024). Security of end-to-end medical images encryption system using trained deep learning encryption and decryption network. Egypt. Inform. J. 28:100541. doi: 10.1016/j.eij.2024.100541

[B20] Khalid I. Shah T. Shah D. Eldin S. M. Asif M. Saddique I. (2022). An integrated image encryption scheme based on elliptic curve. IEEE Access 11, 5483–5501. doi: 10.1109/ACCESS.2022.3230096

[B21] Khatib I. A. Shamayleh A. Ndiaye M. (2024). Healthcare and the internet of medical things: applications, trends, key challenges, and proposed resolutions. Informatics 11:47. doi: 10.3390/informatics11030047

[B22] Kumar M. Mao Y. Wang Y. Qiu T. Chenggen Y. Zhang W. (2017). Fuzzy theoretic approach to signals and systems. Inform. Sci. 418, 668–702. doi: 10.1016/j.ins.2017.08.048

[B23] Long B. Chen Z. Liu T. Wu X. He C. Wang L. (2024). A novel medical image encryption scheme based on deep learning feature encoding and decoding. IEEE Access 12, 38382–38398. doi: 10.1109/ACCESS.2024.3371888

[B24] Luo Y. Ouyang X. Liu J. Cao L. (2019). An image encryption method based on elliptic curve ElGamal encryption and chaotic systems. IEEE Access 7, 38507–38522. doi: 10.1109/ACCESS.2019.2906052

[B25] Manikandan V. Amirtharajan R. (2022). A simple embed over encryption scheme for DICOM images using bülban map. Med. Biol. Eng. Comput. 60, 701–717. doi: 10.1007/s11517-021-02499-4 35040082 PMC8763365

[B26] Mansouri A. Sun P. Lv C. Zhu Y. Zhao X. Ge H. . (2025). Real-time encryption of medical images in IoMT. Knowl. -*Based Syst*. 323:113697. doi: 10.1016/j.knosys.2025.113697

[B27] Masood F. Boulila W. Alsaeedi A. Khan J. S. Ahmad J. Khan M. A. . (2022). A novel image encryption scheme based on Arnold cat map, Newton–Leipnik system and logistic Gaussian map. Multimedia Tools Applic. 81, 30931–30959. doi: 10.1007/s11042-022-12844-w

[B28] Mehmood A. Alawida M. Shafique A. Khan A. N. (2024). Advances and vulnerabilities in modern cryptographic techniques. IEEE Access 12, 27530–27555. doi: 10.1109/ACCESS.2024.3367232

[B29] Ningthoukhongjam T. R. Heisnam S. D. Khumanthem M. S. (2024). Medical image encryption through chaotic asymmetric cryptosystem. IEEE Access 12, 73879–73888. doi: 10.1109/ACCESS.2024.3404088

[B30] Phong L. T. Aono Y. Hayashi T. Wang L. Moriai S. (2017). “Privacy-preserving deep learning: revisited and enhanced,” in Proceedings of the International Conference on Applications and Techniques in Information Security (Singapore: Springer), 100–110. doi: 10.1007/978-981-10-5421-1_9

[B31] Prabhavathi K. Anandaraju M. B. (2023). An efficient medical image encryption algorithm for telemedicine. Microprocess. Microsyst. 101:104907. doi: 10.1016/j.micpro.2023.104907

[B32] Rao Y. Ni J. (2016). “A deep learning approach to detection of splicing and copy-move forgeries in images,” in Proceedings of the IEEE International Workshop on Information Forensics and Security (IEEE), 1–6. doi: 10.1109/WIFS.2016.7823911

[B33] Rasool R. U. Ahmad H. F. Rafique W. Qayyum A. Qadir J. (2022). Security and privacy of internet of medical things: a contemporary review in the age of surveillance, botnets, and adversarial ML. J. Netw. Comput. Applic. 201:103332. doi: 10.1016/j.jnca.2022.103332

[B34] Selvakumar B. Abinaya P. Lakshmanan B. Sheron S. Rajini T. S. (2024). Hybrid image encryption using advanced least significant bit algorithm, chaotic maps and DNA encoding for digital healthcare. J. Intell. Fuzzy Syst. 46, 9139–9153. doi: 10.3233/JIFS-236637

[B35] Umar T. Nadeem M. Anwer F. (2024). Chaos based image encryption scheme for cloud storage. Expert Syst. Applic. 257:125050. doi: 10.1016/j.eswa.2024.125050

[B36] Yasser I. Khalil A. T. Mohamed M. A. Samra A. S. Khalifa F. (2021). A robust chaos-based technique for medical image encryption. IEEE Access 10, 244–257. doi: 10.1109/ACCESS.2021.3138718

[B37] Ye J. Ni J. Yi Y. (2017). Deep learning hierarchical representations for image steganalysis. IEEE Trans. Inform. Forens. Sec. 12, 2545–2557. doi: 10.1109/TIFS.2017.2710946

[B38] Zhang J. Han J. Ye Y. (2021). Multi-image encryption algorithm based on image hash. IET Image Process. 15, 885–896. doi: 10.1049/ipr2.12069

[B39] Zhang X. Di J. Niu Y. (2024). Image encryption scheme based on double permutation and DNA. Multimedia Tools Applic. 83, 57291–57316. doi: 10.1007/s11042-023-17392-5

[B40] Zhang Y. Lin P. Chen J. Sun W. (2026). A hybrid cryptosystem for medical image security: integrating finite fields with conservative hyperchaos. Nonlin. Dyn. 114:129. doi: 10.1007/s11071-025-11994-4

[B41] Zhang Y. Xie H. Sun J. Zhang H. (2022). An efficient multi-level encryption scheme for stereoscopic medical images based on coupled chaotic system and Otsu threshold segmentation. Comput. Biol. Med. 146:105542. doi: 10.1016/j.compbiomed.2022.105542 35483228

[B42] Zhou S. Zhao Z. Wang X. (2022). Novel chaotic colour image cryptosystem with deep learning. Chaos, Solitons Fractals 161:112380. doi: 10.1016/j.chaos.2022.112380

[B43] Zia U. McCartney M. Scotney B. Martinez J. AbuTair M. Memon J. . (2022). Survey on image encryption techniques using chaotic maps. Int. J. Inform. Security 21, 1–19. doi: 10.1007/s10207-022-00588-5

